# Orthopaedic Management of Bone Metastases from Lung Cancer

**DOI:** 10.3390/cancers18193228

**Published:** 2026-10-07

**Authors:** Júlio Martins Lopes, Vânia Oliveira, Pedro Cardoso, Manuel Magalhães, Paula Fidalgo, António Araújo

**Affiliations:** 1 School of Medicine and Biomedical Sciences, ICBAS, University of Porto, 4050-313 Porto, Portugal; vaniacoliveira@gmail.com (V.O.); pffcardoso@gmail.com (P.C.); antonio.araujo@chporto.min-saude.pt (A.A.); 2 Department of Orthopaedics, Unidade Local de Saúde de Santo António, 4050-366 Porto, Portugal; 3 Department of Medical Oncology, Unidade Local de Saúde de Santo António, 4050-366 Porto, Portugal; manuel.magalhaes@gmail.com (M.M.); paulasalesfidalgo@gmail.com (P.F.)

**Keywords:** lung cancer, bone metastases, orthopaedic management, pathological fractures, metastatic spinal cord compression, clinical decision-making

## Abstract

Background/Objectives: Bone metastases from lung cancer can cause pain, pathological fracture, spinal instability, metastatic spinal cord compression and loss of independence. Lung cancer-specific evidence for orthopaedically relevant local management remains fragmented. This review assessed outcomes across spinal, appendicular and pelvic skeletal disease, while explicitly examining the anatomical distribution and limitations of the evidence. Methods: PubMed, Web of Science, Europe PMC and Scopus were searched for original studies published from January 2015 to December 2025. Eligible studies evaluated orthopaedic, surgical, minimally invasive or percutaneous skeletal interventions, including radiotherapy when combined with or directly compared with an orthopaedically relevant local strategy. Two reviewers independently screened studies, extracted data and completed the full Joanna Briggs Institute checklist applicable to each design. Findings were synthesised narratively, with evidence certainty assessed using GRADE. Results: Thirty-three studies comprising 3585 participants with lung cancer were included; 26 were retrospective observational studies. Thirty studies predominantly evaluated axial disease, mainly spinal metastases, whereas only six included appendicular lesions; these categories were not mutually exclusive. Local interventions were associated with pain relief, maintenance or recovery of ambulation, neurological improvement and local symptom control in selected patients. Comparative survival findings were inconsistent and vulnerable to selection bias and confounding by indication; no causal survival benefit of surgery was established. Complication patterns differed by intervention type. Overall certainty was low to very low and was weakest for appendicular and pelvic disease. Conclusions: Orthopaedic management can be clinically important for preserving function and controlling mechanical or neurological complications, but the evidence is substantially stronger for spinal than for extraspinal disease. Treatment should remain multidisciplinary and individualised. The proposed decision frameworks are conceptual and guideline-informed rather than validated treatment algorithms.

## Simple Summary

Bone metastases are a common and serious complication of lung cancer, causing pain, fractures, spinal instability, neurological impairment and loss of mobility and independence. As advances in cancer treatment extend survival for some patients, managing these complications becomes increasingly important. This review examines the role of surgery and minimally invasive procedures in relieving symptoms, preserving function and supporting ongoing cancer treatment, with attention to patient selection, timing and treatment extent. The available evidence predominantly concerns spinal metastases, with limited data on appendicular and pelvic disease. Findings suggest improvements in comfort, mobility and quality of life in selected patients, although a survival benefit has not been established. The predominantly observational evidence and differences between treatment groups warrant cautious interpretation. Care should remain multidisciplinary and individualised, considering prognosis, mechanical stability, neurological risk, functional capacity and patient goals.

## 1. Introduction

Lung cancer remains a leading cause of cancer-related mortality and is frequently diagnosed at an advanced stage. Lung cancer screening programmes using low-dose computed tomography are increasingly being implemented or considered internationally. Nevertheless, metastatic disease remains a major clinical problem [1]. Non-small-cell lung cancer (NSCLC) accounts for approximately 85–90% of cases, whereas small-cell lung cancer (SCLC) is less common and more aggressive; these subtypes differ in biology, treatment options and prognosis. Lung cancer is also among the most frequent primary tumours associated with skeletal metastases, providing the rationale for a lung cancer-specific orthopaedic review [2,3,4].

Lung cancer is characterised by a marked propensity for skeletal dissemination. Bone metastases arise when tumour cells disseminate from the primary lung tumour, survive within the bone microenvironment and establish clinically apparent metastatic lesions, disrupting the balance between bone formation and resorption and promoting skeletal destruction [3,4,5,6]. Bone metastases occur in approximately 20–40% of patients with NSCLC and are particularly common in advanced disease. Within the skeleton, the spine is the most frequently affected site, followed by the ribs and pelvis, whereas the proximal femur and humerus are the most common appendicular locations. Because of its biomechanical and neurological significance, spinal involvement has disproportionate clinical consequences [3,7,8,9].

Skeletal metastases are among the most clinically significant causes of cancer-related pain. Spinal and appendicular metastases may cause pathological fractures, vertebral collapse, mechanical instability, neurological compromise and loss of ambulation, with major consequences for independence and quality of life (QoL). These complications are commonly captured as skeletal-related events (SREs), including pathological fracture, spinal cord compression and the need for surgery or radiotherapy (RT). Among these complications, metastatic spinal cord compression (MSCC) represents an oncological emergency, with motor dysfunction being the second most common presenting feature after pain in spinal metastatic disease. SREs are also associated with greater healthcare resource utilisation and worse survival. This clinical burden is particularly relevant in lung cancer, in which skeletal disease often combines aggressive mechanical destruction with marked prognostic heterogeneity, making extrapolation from mixed-primary metastatic bone series especially problematic [4,10,11,12,13,14,15,16].

Bone metastases from lung cancer were historically associated with poor survival, favouring a predominantly palliative approach. However, advances in systemic therapy and multidisciplinary care have altered the contemporary prognostic landscape, and meaningful functional recovery is now achievable in selected patients [3,17,18,19,20,21,22,23,24]. Consequently, traditional classification-based prognostic tools remain useful but may no longer fully capture outcomes in the era of targeted therapy, immunotherapy and modern multidisciplinary management. In selected patients, effective local orthopaedic treatment may therefore serve not only a palliative function but also a treatment-enabling role by preserving ambulation, autonomy and the functional reserve required for continuation of systemic therapy. These changes support the need for more individualised treatment models that integrate biological, functional and treatment-related variables [18,19,22,25,26,27,28].

For treatment planning, metastatic burden should be distinguished as solitary, oligometastatic or multiple skeletal disease. A solitary bone metastasis refers to a single confirmed metastatic bone lesion. Oligometastatic bone disease denotes a limited metastatic state, commonly comprising up to five lesions potentially suitable for consolidative local treatment. Multiple skeletal metastases indicate multifocal or disseminated bone involvement, usually associated with greater systemic tumour burden and predominantly palliative treatment goals [3,9,29,30,31]. Although surgery remains predominantly palliative in intent, it may be essential in selected patients to relieve pain, restore or preserve mechanical stability, decompress neural structures and maintain ambulation. When appropriate, orthopaedic intervention is most valuable before structural failure or neurological decline becomes established. The appropriate extent of intervention remains controversial, particularly when neural decompression and durable reconstruction must be balanced against operative morbidity and expected survival [12,15,18,27,32,33]. In spinal metastatic disease, tools such as the Spinal Instability Neoplastic Score (SINS) may assist decision-making when interpreted in conjunction with neurological status and overall prognosis [34]. In this context, timing becomes a determinant of orthopaedic value, because delayed intervention may allow preventable mechanical failure, neurological deterioration or loss of functional reserve to become irreversible [33,35,36,37].

Minimally invasive and hybrid local therapies have further expanded treatment options, particularly in frail patients, in previously irradiated lesions and when the primary aim is palliation [38,39,40,41,42,43].

Although spinal disease dominates the clinical discussion, appendicular metastases remain clinically important because they threaten weight-bearing capacity, upper-limb function, mobility and independence. Their management raises distinct questions regarding fracture prevention, reconstruction, implant durability and proportionality of intervention [7,13,21,44,45,46,47,48,49].

Despite major advances in systemic and local treatment, important evidence gaps persist regarding the orthopaedic management of bone metastases from lung cancer. Much of the available literature pools different primary tumours, skeletal sites and treatment indications, whereas lung cancer-specific orthopaedic data remain limited and are often confined to retrospective, modality-specific or highly selected cohorts. As a result, the role of open surgery, minimally invasive fixation, ablative procedures and hybrid approaches remains incompletely defined in this population [13,22,23,38].

From an orthopaedic perspective, bone metastases from lung cancer should be approached as time-sensitive mechanical and neurological problems rather than as imaging findings alone [6,16,50,51]. Against this background, this systematic review aims to evaluate the clinical impact of surgical and minimally invasive orthopaedic interventions in patients with bone metastases from lung cancer, compared with non-surgical or alternative strategies, with emphasis on pain control, functional and neurological outcomes, complications, survival and factors relevant to treatment selection in contemporary practice [12,18,19].

## 2. Materials and Methods

This systematic review was conducted in accordance with the Preferred Reporting Items for Systematic Reviews and Meta-Analyses (PRISMA) 2020 statement [52].

The aim was to evaluate the available evidence on surgical and minimally invasive orthopaedic management strategies for bone metastases from lung cancer, with particular emphasis on pain control, functional and neurological outcomes, complications, survival and treatment selection.

Study selection and data extraction were performed independently by two reviewers using predefined eligibility criteria.

### 2.1. Eligibility Criteria

Original studies evaluating adults with bone metastases from lung cancer were eligible. Mixed-primary cohorts were included only when patients with lung cancer formed a clearly identifiable subgroup and relevant outcome data could be extracted separately for that subgroup. Eligible designs included randomised trials, prospective or retrospective cohort studies, case-control studies and case series with more than five patients. Studies had to be published in English between January 2015 and December 2025 and report orthopaedically relevant outcomes after surgical, minimally invasive or percutaneous local skeletal interventions. Radiotherapy-based studies were eligible when RT was combined with, delivered after, or directly compared with an orthopaedically relevant local skeletal intervention. Radiotherapy-only studies were excluded unless they directly compared radiotherapy with an orthopaedically relevant local skeletal intervention. Paediatric studies, non-pulmonary metastases, primary bone tumours, haematological malignancies, purely systemic oncological studies, reviews, editorials, conference abstracts, case reports, animal or in vitro studies and case series with five or fewer patients were excluded. Reports from the same centre or potentially overlapping populations were retained only when they provided distinct, non-duplicative outcomes or addressed different clinically relevant questions. When multiple reports were derived from the same cohort, participants were counted only once in the aggregated clinical sample.

Where supported by the individual databases, predefined restrictions relating to publication year, English language, human studies, adult populations, abstract availability and publication type were applied at the database-search stage before records were exported for deduplication and screening. Eligibility criteria that could not be reliably implemented through database filters were assessed during title/abstract and full-text screening.

### 2.2. Information Sources and Search Strategy

PubMed, Web of Science, Europe PMC and Scopus were searched, with the final search performed on 31 December 2025. The electronic strategy was intentionally anchored to lung cancer, skeletal metastases and orthopaedic/mechanical local treatment concepts. Consequently, RT was captured when it appeared within an orthopaedically relevant comparative or multimodal study rather than as a standalone RT literature search. Search terms were adapted to each database. PubMed yielded the highest number of records and the search strategy used for this database is presented below: ((Lung Neoplasms OR Carcinoma, Non-Small-Cell Lung OR Carcinoma, Small Cell OR Lung cancer OR pulmonary cancer OR pulmonary neoplasm*)) AND ((Bone Neoplasms/secondary OR bone metast* OR skeletal metast* OR long bone* metast* OR spinal metast* OR vertebral metast* OR femur metast* OR humeral metast* OR tibia metast* OR Fractures, spontaneous OR pathologic fracture* OR impending fracture OR vertebral fracture*)) AND ((Orthopaedic Procedures OR orthop* OR spinal surgery OR spine surgery OR laminectomy OR corpectomy OR fracture fixation OR intramedullary nail* OR arthroplast* OR endoprosthe* OR megaprosthe* OR prosthetic replacement OR tumour prosthesis OR modular endoprosthesis OR bone plate* OR vertebroplasty OR kyphoplasty OR cementoplasty OR bone cement OR spinal fusion OR separation surgery OR bone stabilization OR spinal stabilization OR internal fixation OR screw fixation OR percutaneous fixation)). The corresponding search strategies for Web of Science, Europe PMC and Scopus are provided in Appendix A. Ethical approval was not required because all data were obtained from published studies.

**Figure 1:**
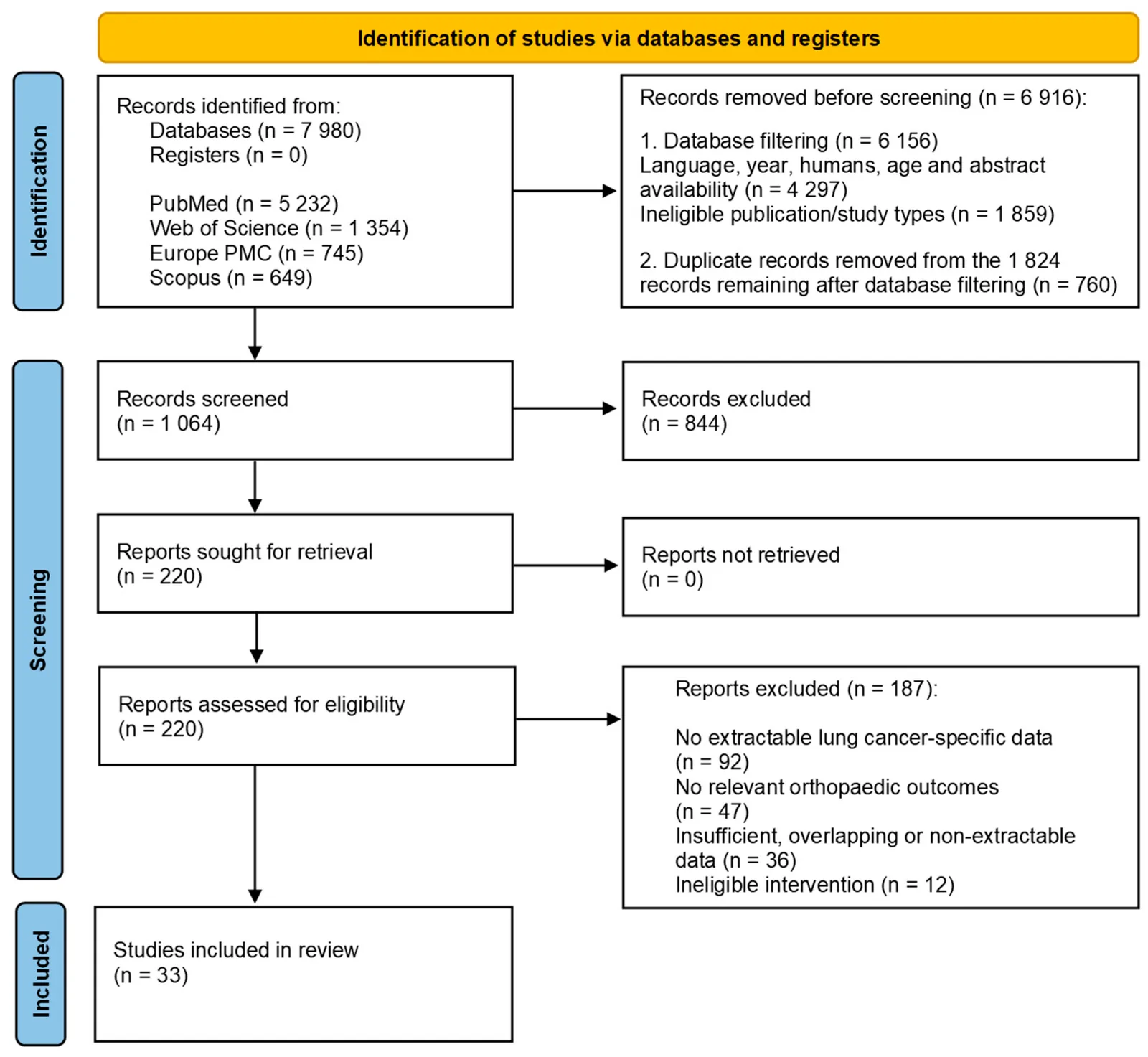
PRISMA 2020 flow diagram of study identification and selection.

A total of 7980 records were identified through the database searches. Before deduplication, 6156 records were removed through database-specific filtering: 4297 on the basis of language, publication year, human studies, age, and abstract availability, and 1859 on the basis of ineligible publication or study type. The remaining 1824 records were imported into Mendeley Reference Manager (Elsevier, Amsterdam, The Netherlands), which automatically identified and removed 760 duplicates. This left 1064 unique records for title and abstract screening. Of these, 844 were excluded because they did not meet the predefined eligibility criteria. The remaining 220 reports were sought and successfully retrieved for full-text assessment. Following full-text assessment, 187 reports were excluded: 92 because lung cancer-specific data could not be extracted, 47 because they did not report relevant orthopaedic outcomes, 36 because relevant outcome data were insufficient, could not be extracted for synthesis, or because the report contained overlapping data without additional eligible information, and 12 because the intervention was outside the predefined scope of the review. Ultimately, 33 studies met the eligibility criteria and were included in the review. The PRISMA flow diagram (Figure 1) reproduces this sequence and these numbers. The completed PRISMA 2020 checklist and PRISMA 2020 for Abstracts checklist are provided as Supplementary Tables S1 and S2 and the detailed study characteristics and outcome data are provided in Supplementary Table S3.

This systematic review was registered in PROSPERO, the International Prospective Register of Systematic Reviews, under registration number CRD420261380000. The registered review protocol is publicly accessible at: https://www.crd.york.ac.uk/PROSPERO/view/CRD420261380000 (accessed on 22 August 2026).

### 2.3. Quality Assessment

Methodological quality was assessed using the appropriate Joanna Briggs Institute Critical Appraisal Checklist for each study design (cohort or case-control) [53]. The complete checklist was applied to every eligible study of the corresponding design: all 11 cohort items were assessed for both prospective and retrospective cohort studies, and all 10 case-control items were assessed for the case-control study. Each item was rated as “Yes”, “No”, “Unclear” or “Not applicable”. Item-level responses are retained in Supplementary Tables S4 and S5 and interpreted with emphasis on participant selection and comparability, exposure and outcome ascertainment, identification and management of confounding, adequacy and completeness of follow-up, and appropriateness of statistical analysis. Two reviewers independently completed the assessments, and disagreements were resolved by consensus. Methodological quality informed interpretation of the evidence but was not used as a criterion for study exclusion.

### 2.4. Data Extraction and Synthesis

Extracted data included study design, sample size, lung cancer subtype, metastatic site and burden, fracture or instability status, intervention, comparator, pain, functional and neurological outcomes, quality of life (QoL), local progression, complications, reoperation, follow-up, survival, effect estimates when reported, and prognostic factors. Owing to substantial clinical and methodological heterogeneity, no meta-analysis was performed. Findings were synthesised narratively by clinically relevant domains.

The certainty of evidence for each outcome was assessed using the Grading of Recommendations Assessment, Development and Evaluation (GRADE) approach [54]. For intervention outcomes, observational evidence was initially rated as low certainty, with downgrading or upgrading where justified according to GRADE criteria. Prognostic associations were assessed using prognostic-factor GRADE guidance, with observational evidence initially rated as high certainty [55]. The assessment was conducted without meta-analysis. Outcome-specific certainty ratings and the rationale for each judgement are presented in Supplementary Table S6.

## 3. Results

Thirty-three studies comprising 3585 participants with lung cancer were included. The two Lei et al. [56,57] reports were derived from the same 73-patient cohort, which was counted once in the aggregated clinical sample. The population-based SEER component of Hu et al. [58] was not included in this total; only the FUSCC clinical cohort contributed orthopaedic outcome data. Most included studies were retrospective observational studies (26/33), including one retrospective case-control study, whereas six were prospective cohorts. One study used a mixed retrospective–prospective design. Follow-up was commonly 12–24 months, ranging from short-term postoperative assessment to a median of 47.4 months in Zhai et al. [59]. Interventions included open decompression and stabilisation, vertebral augmentation, thermal ablation, cement-based reinforcement, radiotherapy-based strategies and multimodal local treatment. Table 1 and Table 2 summarise the 33 studies, separated into spinal-predominant and appendicular/pelvic or mixed-site evidence to make the anatomical imbalance explicit. Detailed study characteristics are provided in Supplementary Table S3.

### 3.1. Study and Disease Profile

#### 3.1.1. Axial Versus Appendicular Skeleton

Of the 33 included studies, 30 evaluated predominantly axial skeletal metastatic disease, with spinal metastases constituting the main anatomical focus. Six studies also included appendicular skeletal lesions, particularly long-bone involvement. These categories were not mutually exclusive. Non-spinal axial sites, including the pelvis and ribs, were mainly represented within broader skeletal metastasis cohorts.

Most reviewed studies focused on vertebral metastases or MSCC. Within spinal series, thoracic involvement was generally the most frequent site, followed by lumbar disease, whereas cervical and sacral lesions were less common [58,66].

Appendicular disease was less frequently studied and, when reported, most commonly involved the femur and humerus. In the broader skeletal metastasis cohort reported by Cho et al. (2019) [9], the spine was the most frequent site (59.9%), followed by the pelvis, ribs, femur and humerus. In the oligometastatic cohort of Congedo et al. (2022) [83], anatomical distribution was more balanced, although vertebral lesions remained the single most frequent site.

#### 3.1.2. Single/Oligo Versus Multiple Metastases

Reporting of solitary, oligometastatic and multiple skeletal disease was inconsistent across studies, and most studies did not stratify treatment according to metastatic burden. When reported, multiple skeletal lesions were generally more common than solitary disease, except in selected oligometastatic cohorts. Congedo et al. (2022) [83], included only patients with synchronous bone-oligometastatic NSCLC, predominantly with solitary bone involvement, within a multimodal radical-intent strategy: bone lesions were treated surgically when radical local resection was feasible, whereas non-surgically managed lesions received local RT. By contrast, Hu et al. (2022) [58] and Xu et al. (2021) [80] reported a predominance of multiple bone or spinal metastatic sites in surgical cohorts. In the mixed appendicular cohort of Cho et al. (2019) [9], 128 patients (63.4%) already had multiple distant metastases, including skeletal metastases at lung cancer diagnosis, whereas 35 patients (17.3%) had skeletal metastases only. In the broader spinal or skeletal cohorts, metastatic burden was mainly reported as a disease descriptor or prognostic variable, and treatment was selected according to the dominant local indication, including pain, spinal instability, neurological compression, vertebral collapse or impending/established pathological fracture, rather than metastatic burden alone.

#### 3.1.3. Impending Versus Established Fractures

The distinction between established and impending fractures was more clearly reported in appendicular studies than in spinal cohorts. In long-bone metastases, impending fracture was usually defined using mechanical risk stratification, most commonly the Mirels score. Kim et al. (2016) [84] included patients with femoral or humeral metastases and a Mirels score >9, although the exact proportion of impending versus established fractures was not reported. Cho et al. (2019) [9] grouped pathological and impending fractures together as indications for orthopaedic surgery. In spinal cohorts, structural compromise was more often reported as vertebral fracture, collapse, instability or neural compression than as a binary classification of impending versus established fracture [58,70,81].

#### 3.1.4. Surgical Techniques

Surgical strategies ranged from palliative decompression and stabilisation to more extensive excisional procedures, as well as minimally invasive vertebral augmentation and ablation-based techniques. Across the 33 included studies, 21 (63.6%) evaluated open surgical approaches, 5 (15.2%) focused on minimally invasive techniques and 3 (9.1%) compared surgical with non-surgical strategies, while the remaining studies involved mixed or multimodal treatment frameworks.

Open spinal surgery was the most frequently reported local treatment approach, particularly in the setting of MSCC or mechanical instability [64,80,81]. Within open surgery, posterior decompression with instrumented stabilisation was the most commonly reported approach, although the extent of resection varied from laminectomy to corpectomy and, in selected cases, spondylectomy/total vertebrectomy. Park et al. (2016) [71] and Jung et al. (2021) [64], for example, reported this range of procedures.

Minimally invasive spinal studies mainly involved PVP, either alone or combined with MWA, whereas extraspinal studies involved ablation plus osteoplasty/cementoplasty. In appendicular disease, cement augmentation was used as an adjunct to intramedullary nailing (IMN) rather than as a standalone spinal procedure. Liu et al. (2023) [70] and Wu et al. (2024) [79] illustrate the increasing use of combined percutaneous spinal approaches using MWA plus PVP, whereas Wei et al. (2015) [86] applied a comparable ablation–osteoplasty strategy in extraspinal lesions.

Several studies reported more than one local treatment modality within the same cohort. Wang et al. (2021) [85] reported local treatment with surgery, percutaneous osteoplasty and RT within a multidisciplinary setting. Zhang et al. (2016) [36], Yang et al. (2017) [81] and Tang et al. (2016) [76] reported comparisons between surgery, RT alone, conservative management or other non-surgical strategies.

### 3.2. Clinical Outcomes

#### 3.2.1. Pain Relief

Pain outcomes were reported across most treatment categories. Reductions in Visual Analogue Scale (VAS) or Numeric Rating Scale (NRS) scores were reported after open spinal surgery, vertebral augmentation, thermal ablation combined with vertebroplasty or osteoplasty, cement-augmented intramedullary nailing, RT-based strategies and MDT-guided local treatment. Some studies also reported reduced analgesic requirements. Baseline pain was typically in the moderate-to-severe range, often around VAS 6–8, and generally decreased to mild or low-to-moderate levels within days or weeks after treatment. Jung et al. (2021) [64] reported significant improvement in pain scores after laminectomy, corpectomy and spondylectomy, with reductions maintained at follow-up across all groups.

Comparative interventional studies reported differences between treatment modalities. Liu et al. (2023) [70] showed that PVP alone and MWA plus PVP achieved similar short-term pain relief, whereas, at 24 weeks, pain scores and daily morphine consumption were lower in the combined-treatment group. In appendicular metastases, Kim et al. (2016) [84] reported that cement-augmented IMN provided greater pain reduction than conventional IMN, with lower immediate postoperative VAS scores (3.8 vs. 6.0; *p* < 0.001) and lower follow-up VAS scores (3.3 vs. 6.6; *p* < 0.001). In the prospective MDT-guided cohort of Wang et al. (2021) [85], surgery, osteoplasty and RT all produced significant pain reduction, without significant between-modality differences. Zhong et al. (2022) [82] reported lower short-term pain scores after posterior decompression and fixation combined with intraoperative MWA than after decompression and fixation alone.

#### 3.2.2. Functional and Neurological Recovery

Functional and neurological outcomes were reported using heterogeneous measures, including Frankel grade, ASIA grade, ECOG performance status, Karnofsky Performance Status (KPS), Barthel Index (BI), ambulatory status and Oswestry Disability Index (ODI). Surgical decompression and stabilisation were frequently followed by improvement or preservation of neurological status, ambulation and broader functional measures.

Across spinal surgery cohorts, preservation or recovery of ambulation was one of the most frequently reported postoperative outcomes [56,57,66,75]. Park et al. (2016) [71] reported recovery of ambulation in 59.0% of previously non-ambulatory patients and improvement of at least one ECOG grade in 66.0% of the cohort. 

Zhai et al. (2023) [59] reported postoperative improvement in KPS in most patients with poor baseline status, and complete decompression was associated with better postoperative neurological and functional outcomes than palliative surgery. Wang et al. (2021) [85] reported improved QoL and functional outcomes across surgery, osteoplasty and RT.

In selected non-randomised comparative cohorts, surgery was associated with better neurological or functional outcomes than non-surgical strategies. Zhang et al. (2016) [36] reported better Frankel and KPS outcomes after surgery combined with RT than after RT alone. Park et al. (2023) [72] reported neurological deterioration only in the RT-alone group in a propensity-matched comparison. Lin et al. (2020) [69] reported neurological improvement in 67% of patients presenting with deficits and postoperative ambulatory status in 95.4% of the cohort after spinal stabilisation.

#### 3.2.3. Local Tumour Progression

Local tumour progression was reported in a limited number of studies, mainly in minimally invasive, cement-augmentation and selected surgical cohorts. Liu et al. (2023) [70] reported lower local tumour progression after MWA plus PVP than after PVP alone (7.14% vs. 33.0%; *p* = 0.02). In multivariate analysis, MWA plus PVP was identified as an independent protective factor for local tumour progression (*p* = 0.028). Wu et al. (2024) [79] reported no local tumour progression on follow-up imaging after CT-guided MWA plus PVP. In appendicular metastases, Kim et al. (2016) [84] reported greater suppression of local tumour progression with cement-augmented IMN than with conventional IMN (50% vs. 8%; *p* = 0.022). In open spinal surgery cohorts, local progression was more commonly reported through revision surgery for progression at the index level or new spinal metastatic involvement. Jung et al. (2021) [64] reported revision surgery in 19.0% of the laminectomy group, 25.0% of the corpectomy group and 7.7% of the spondylectomy group. In the oligometastatic cohort of Congedo et al. (2022) [83], local recurrence was reported in 29.6% of patients.

#### 3.2.4. Quality of Life

QoL outcomes were reported using heterogeneous instruments across studies. Tang et al. (2016) [76] used the FACT-G questionnaire at baseline and at 1, 3, 6 and 9 months, reporting higher total, physical, emotional and functional well-being scores in the surgery group at each follow-up time point than at baseline and in the non-surgical group (*p* < 0.001). Zhang et al. (2016) [36] used the EORTC QLQ-C30 and reported greater improvement after surgery combined with RT than after RT alone (*p* = 0.004). Gao et al. (2020) [62] assessed QoL using the EQ-5D-3L and reported an increase in mean utility score after surgery for SCLC-related spinal cord compression, from 0.3394 preoperatively to 0.5884 postoperatively.

Li et al. (2015) [67] reported improved activities of daily living (ADL) after PVP plus intensity-modulated RT, with scores increasing from 50.9 ± 11.7 before treatment to 82.3 ± 9.9 after treatment. Wang et al. (2021) [85] reported improved QoL scores after multidisciplinary team-guided local treatment, including surgery, osteoplasty and RT. Wu et al. (2024) [79] reported significant improvement in ODI scores after MWA plus PVP (*p* < 0.05).

#### 3.2.5. Survival Outcomes

Survival outcomes varied across studies, reflecting heterogeneity in patient selection, disease burden and treatment strategy. In most spinal series, median overall survival after diagnosis or surgery ranged from approximately 4.6 to 16 months, although longer survival estimates were reported in selected subgroups with more favourable tumour biology or lower metastatic burden. Amelot et al. (2020) [60], Rothrock et al. (2021) [73] and Park et al. (2016) [71] reported median survival of 5.9, 4.6 and 5.2 months, respectively, whereas Zhai et al. (2023) [59] reported a median survival of 16 months and Takahashi et al. (2025) [74] reported 6-month and 1-year survival rates of 73.1% and 56.3%.

Findings regarding surgery and survival were mixed. Some analyses found no independent survival advantage for surgery after adjustment for confounding factors. Other studies, including Tang et al. (2016) [76], Yang et al. (2017) [81] and Cho et al. (2019) [9], reported longer survival among selected operated patients.

Longer survival estimates were reported in the oligometastatic cohort of Congedo et al. (2022) [83]. In patients with synchronous bone-oligometastatic NSCLC treated with multimodal radical-intent therapy, 1-year and 5-year overall survival were 96% and 38%, respectively. Surgical treatment of bone lesions was associated with longer disease-free survival than non-surgical local treatment in this selected cohort. Because treatment occurred within a multimodal strategy in a biologically favourable subgroup, the observed survival difference cannot be attributed to surgery alone.

#### 3.2.6. Complications and Safety

Complication rates varied according to procedure type, case selection and reporting methodology. In open surgical series, reported complications included wound, pulmonary, vascular and neurological complications, as well as perioperative mortality. Truong et al. (2020) [77] reported 30-day mortality of 4.6%. Zhai et al. (2023) [59] reported complications in 22.3% of patients, including both mild and severe events, with pulmonary complications reported among the severe events.

In minimally invasive series, reported adverse events mainly included cement leakage, haematoma, infection and procedure-related neurological injury. Cement leakage was frequently asymptomatic in vertebral augmentation studies. Wu et al. (2024) [79] reported cement leakage in 46.8% of treated vertebral levels after MWA plus PVP. Liu et al. (2023) [70] reported lower cement leakage and fewer overall complications with MWA plus PVP than with PVP alone. Wei et al. (2015) [86] reported no peri-procedural deaths after MWA plus osteoplasty, despite an overall complication rate of 28%.

In appendicular metastases, Kim et al. (2016) [84] reported complication rates of 9% with cement-augmented IMN and 15% with conventional IMN, without an increase in complications in the cement-augmentation group. Li et al. (2015) [67] reported asymptomatic cement leakage in three patients after PVP plus intensity-modulated radiotherapy (IMRT), with no severe complications. Congedo et al. (2022) [83] reported 37% minor complications and one major complication, acute respiratory distress syndrome, with no perioperative mortality. Taken together, the reported complication patterns differed by intervention type: open surgery carried wound, pulmonary, thromboembolic, neurological and early mortality risks. Minimally invasive vertebral or ablation/cement procedures were characterised mainly by cement leakage and other procedure-specific events, often asymptomatic. Studies showed substantial heterogeneity in definitions, denominators, patient selection, and follow-up, precluding direct numerical comparison across intervention types.

#### 3.2.7. Prognostic Factors

Reported prognostic factors included performance status, neurological status, systemic disease burden, tumour biology and treatment responsiveness. Amelot et al. (2020) [60] reported that good World Health Organization performance status was independently associated with longer survival. Tang et al. (2015) [75] identified ECOG performance status, preoperative and postoperative Frankel score, postoperative RT and targeted therapy as independent prognostic factors. Takahashi et al. (2025) [74] found that higher vitality and mobility scores were independently associated with longer postoperative survival.

Neurological status was also reported as a prognostic factor. Better Frankel grade, preserved ambulation and shorter duration of motor decline before intervention was associated with longer survival and better postoperative recovery in several studies. Amelot et al. (2020) [60] found that Frankel D and Frankel E grades were independently associated with improved survival. Xu et al. (2021) [80] showed that preoperative Frankel score was an independent prognostic factor in patients undergoing total vertebrectomy, while Zhai et al. (2023) [59] showed that postoperative Frankel grade and postoperative KPS were significantly associated with survival.

Actionable molecular alterations and access to targeted systemic therapy were repeatedly reported in association with longer survival. Amelot et al. (2020) [60] identified EGFR positivity as an independent survival predictor. Chen et al. (2021) [61] incorporated targeted therapy into a survival nomogram, and Zhai et al. (2023) [59] found that postoperative molecular targeted therapy was the only independent predictor of longer survival on multivariate analysis. Park et al. (2023) [72] reported that absence of a targetable mutation was associated with poorer survival in a matched comparison.

Greater systemic disease burden, particularly visceral metastatic involvement and multiple bone lesions, was associated with poorer survival. Chen et al. (2021) [61] incorporated visceral metastasis and extraspinal bone metastases into their model. Visceral metastases were included as adverse prognostic variables in lung cancer-specific scoring systems. Xu et al. (2021) [80] reported that visceral metastasis, elevated D-dimer and higher neutrophil-to-lymphocyte ratio independently predicted poorer survival, while Li et al. (2025) [68] and Jung et al. (2021) [64] reported the negative impact of multiple metastatic sites.

Chen et al. (2021) [61] reported that the revised Tokuhashi score underestimated life expectancy. Huang et al. (2024) [63] reported limited predictive accuracy of the Tomita, revised Tokuhashi and Tokuhashi lung scores, with the latter underestimating overall survival by approximately 35–40%. Wang et al. (2021) [85] reported high predictive performance of a machine-learning decision-tree model, with area under the curve (AUC) values up to 0.92 for identifying patients likely to benefit from local treatment.

## 4. Discussion

This systematic review synthesised the available lung cancer-specific evidence on orthopaedic management of skeletal metastases. Most of the 33 studies evaluated axial, particularly spinal disease, whereas appendicular and pelvic lesions were substantially under-represented. Accordingly, the clinical inferences are more strongly supported for spinal metastases. Extraspinal conclusions are based on fewer, smaller and more heterogeneous studies and should be regarded as lower-certainty associations rather than directly generalisable treatment effects. Interpretation across all sites is further limited by heterogeneity in intervention type, patient selection and outcome reporting [58,59,66].

External guideline recommendations are relevant when translating this evidence into practice. International guidance recognises bone RT as a strategy for pain control and for the prevention or postoperative management of pathological fracture and spinal cord compression, while MSCC is treated as an emergency requiring prompt corticosteroid therapy, urgent imaging and directed intervention. NICE NG234 further emphasises rapid MSCC pathway activation, whole-spine MRI within 24 h, early specialist assessment of spinal stability and multidisciplinary planning before invasive treatment. For appendicular and pelvic metastatic bone disease, BOA/BOOS standards emphasise early multidisciplinary discussion, appropriate staging, diagnostic caution in solitary or atypical lesions, mechanically durable reconstruction and constructs that allow immediate weight-bearing and outlast the patient’s expected survival [6,16,50,51,87,88]. These recommendations are external guidance and should not be interpreted as outcomes generated by the included lung cancer-specific studies. Within this contextual framework, the orthopaedic role includes recognition of mechanical and neurological risk, diagnostic safety, selection of durable reconstruction and integration of local treatment with RT, systemic therapy, rehabilitation and palliative care [16,35,50,51,89]. These principles are summarised in the proposed orthopaedic assessment framework (Supplementary Table S7).

**Figure 2:**
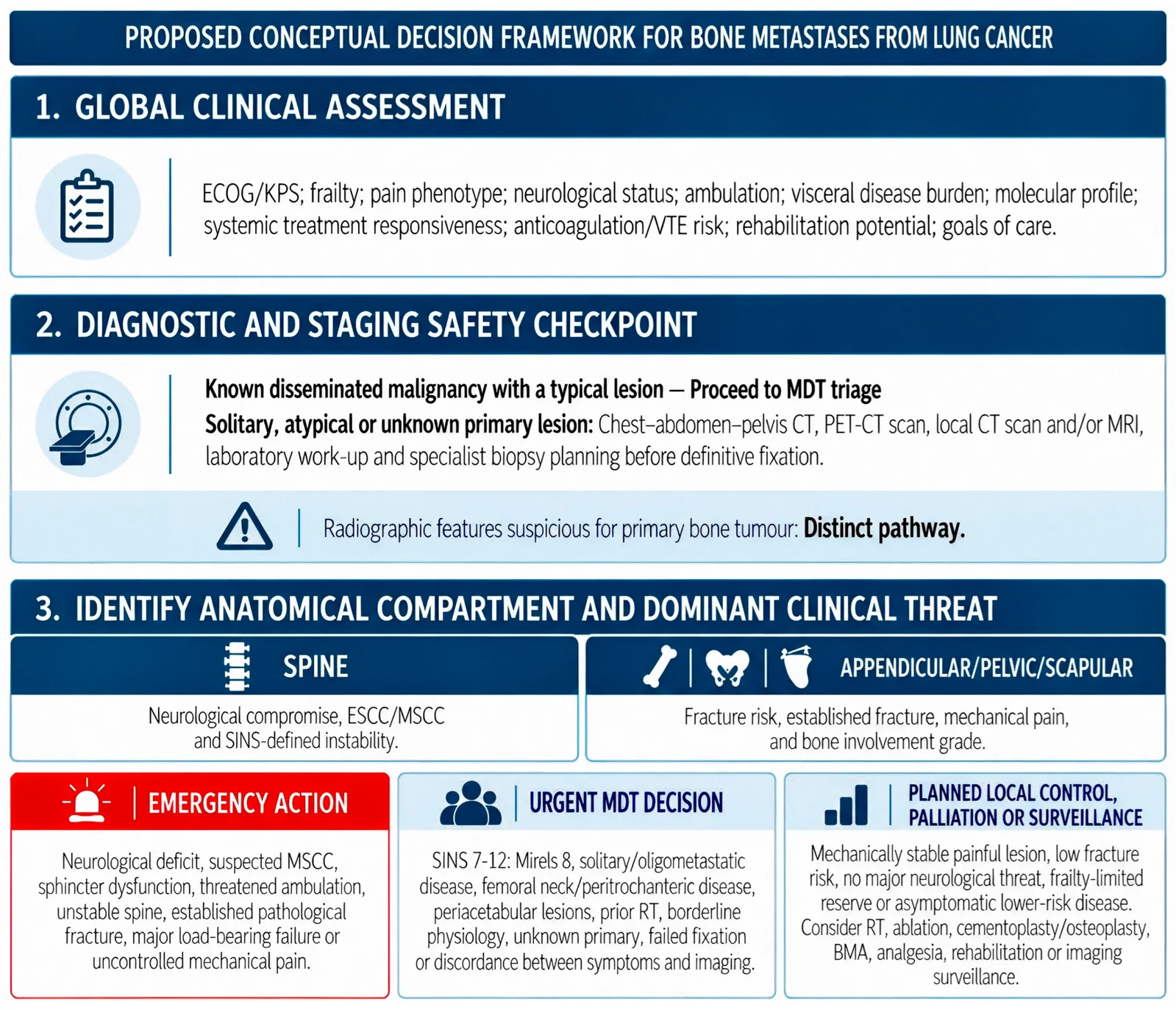
Proposed conceptual decision framework for bone metastases from lung cancer. The framework integrates neurological and mechanical risk, functional reserve, prognosis, anticipated benefit and morbidity of local treatment, diagnostic safety, MSCC escalation, fracture-risk assessment, and multidisciplinary referral. Its structure reflects author synthesis and expert consensus and should be regarded as conceptual rather than prescriptive [6,16,50,51].

Within the included lung cancer-specific studies, treatment selection most consistently reflected the dominant clinical problem rather than the procedure category itself: neurological compromise, mechanical instability or fracture risk, biological prognosis, functional reserve, response to previous treatment and feasibility of ongoing oncological therapy. This perspective is particularly relevant in contemporary lung cancer, where prognosis is heterogeneous and the purpose of local skeletal treatment often extends beyond immediate symptom palliation to preservation of mobility and treatment capacity. These observations support a proportionality-based approach in which expected local benefit, physiological burden and required durability are considered together. Timing may also affect value because delayed intervention can allow neurological deterioration, loss of ambulation or mechanical failure to become irreversible [18,56,58,85]. The integration of these review-derived observations with external guideline safety principles is shown as a proposed conceptual decision framework in Figure 2.

Across the reviewed series, local treatment was most consistently associated with pain relief, preservation or restoration of ambulation, improvement in performance status, reinforcement of structural stability and local symptom control. These associations were strongest and most repeatedly reported in spinal cohorts. Survival, by contrast, was more consistently associated with tumour biology, systemic disease burden, neurological status, visceral metastatic burden, molecular profile and access to effective systemic therapy rather than on the orthopaedic procedure itself. Comparative studies did not consistently demonstrate an independent survival advantage for surgery over RT or other local strategies, and longer survival in some operated cohorts was not clearly separable from selection effects [18,36,60,69,76,85].

In the reviewed spinal series, the clearest indications for surgery arose in the setting of progressive neurological deficit, epidural compression, vertebral collapse or spinal instability. Open surgery remained the dominant treatment approach in such contexts, particularly in MSCC and mechanically unstable vertebral disease. Posterior decompression with instrumented stabilisation was the most frequently reported strategy, whereas more extensive procedures, including corpectomy and spondylectomy, were reserved for selected patients in whom greater local control or more durable reconstruction was required. Importantly, the evidence does not support a simplistic dichotomy between “limited” and “aggressive” surgery. More extensive procedures may offer improved local control or lower revision rates in carefully selected cases, but they do so at the cost of greater operative burden and without consistent evidence of superior survival. Operative extent should therefore be determined by the severity of neurological and mechanical compromise, the durability required, the patient’s broader oncological trajectory and whether the physiological cost of surgery remains proportionate to the expected functional gain and anticipated survival [59,61,64,66,71,80]. The same evidence indicates that not all vertebral lesions require open reconstruction. Stable spinal metastases without major neural compromise were often managed with RT and/or minimally invasive local strategies, particularly when the dominant objective was rapid pain palliation with lower physiological burden. Vertebral augmentation, mainly PVP, with or without thermal ablation, was associated with rapid pain reduction and local control in selected lesions. These techniques appear most appropriate when the goal is pain relief and mechanical reinforcement rather than direct decompression [67,70,79,81]. However, they should not be regarded as substitutes for open surgery in the presence of major instability, deformity or epidural compression. Cement leakage remains common, even when asymptomatic, and embolic complications should remain part of risk assessment, particularly in highly vascular lesions [67,70,79]. When mechanical instability coexists with epidural disease or neurological compromise, surgical decompression combined with instrumented stabilisation should be considered within a multidisciplinary framework. In selected patients, this may take the form of separation surgery, aiming to achieve circumferential neural decompression and restore a safe margin around the spinal cord before postoperative RT or SBRT [16,73,90].

**Figure 3:**
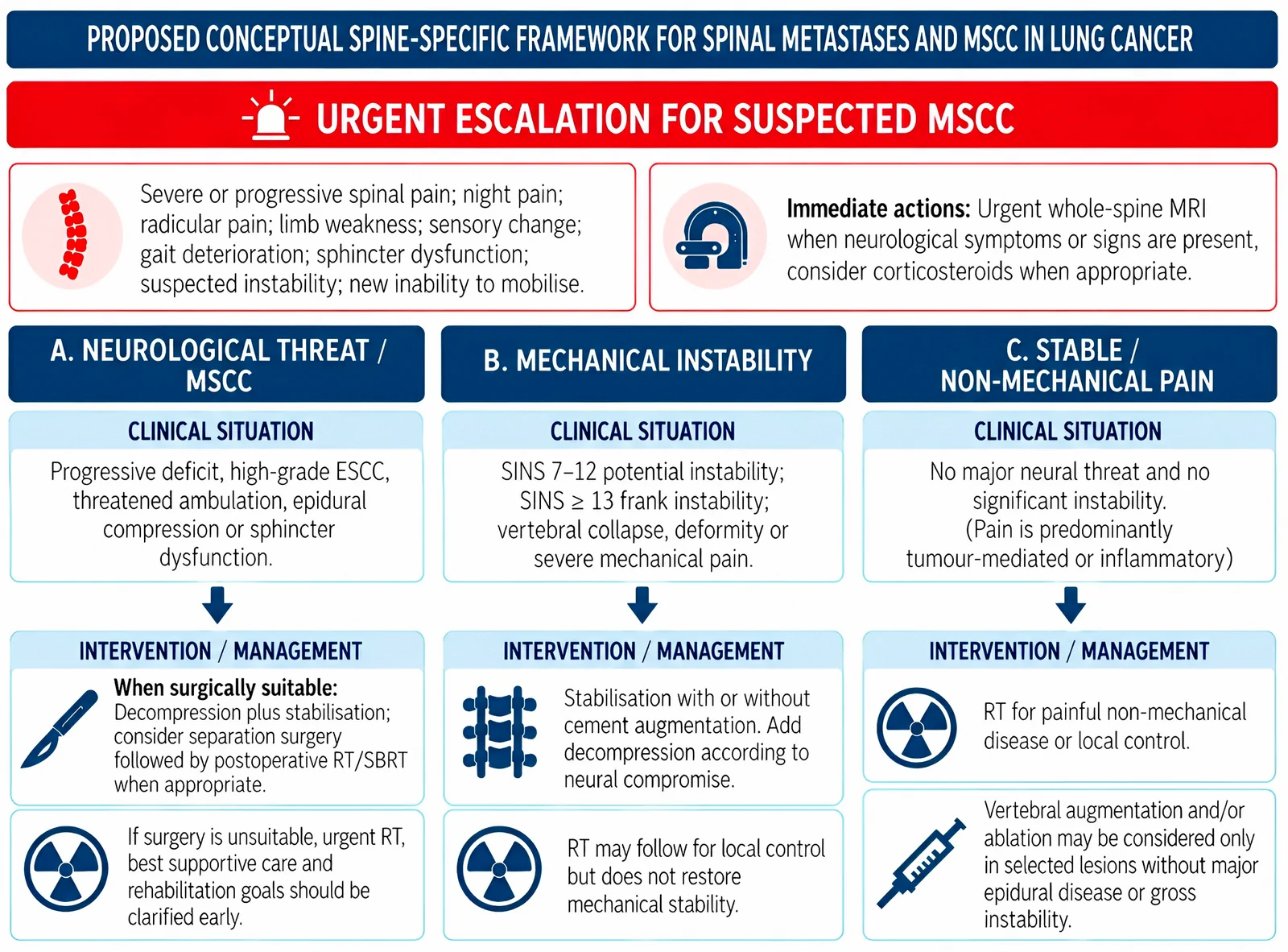
Proposed conceptual spine-specific decision framework for spinal metastases and MSCC in lung cancer. The framework integrates neurological status, spinal stability, local treatment considerations, urgent MRI, corticosteroid consideration, SINS-informed assessment, and radiotherapy principles, with attention to pain, ambulation, neurological function, and local control. Its structure reflects author synthesis and expert consensus and should be regarded as conceptual rather than prescriptive [6,16,50,51].

For spinal disease, the included review evidence supports attention to neurological compromise, mechanical instability and preservation of ambulation. The urgent diagnostic and treatment steps that follow are primarily derived from external MSCC guidance. Suspected MSCC should prompt urgent pathway activation, whole-spine MRI within 24 h and early multidisciplinary discussion. Corticosteroid therapy should be considered promptly in patients with neurological symptoms or signs of MSCC, unless contraindicated, while definitive treatment is being planned. The surgical question is whether decompression and/or stabilisation can plausibly halt neurological decline, relieve mechanical pain, permit mobilisation and allow subsequent oncological treatment. RT alone may be appropriate when the spine is mechanically stable, neurological deficit is absent or non-progressive, or the patient is not a surgical candidate. However, RT does not correct mechanical instability. Persistent mechanical pain, deformity, vertebral collapse or SINS-defined instability therefore warrants specialist surgical assessment [6,16,50,51]. Figure 3 presents these review observations and guideline-derived urgent steps as a spine-specific conceptual framework.

Appendicular and pelvic disease require separate consideration because their orthopaedic objectives differ from those of spinal metastases. In long bones, the main objectives are to preserve load-bearing capacity, limb function and mobility while relieving pain and preventing fracture. Lung cancer-specific evidence is sparse in this setting. In the available studies, cement-augmented intramedullary fixation was associated with greater pain relief and lower local tumour progression than conventional nailing in selected femoral or humeral metastases, while ablation–osteoplasty/cementoplasty strategies were associated with rapid pain relief and local control in selected extraspinal lesions [9,49,84,86]. Oliveira et al. (2021) [49] reported rapid pain relief and functional improvement after local radiofrequency ablation and cementing combined with internal fixation for long-bone metastases, illustrating a potential hybrid strategy when pain control, mechanical reinforcement and early functional recovery are treatment objectives. These observations should be interpreted cautiously because the appendicular evidence base is much smaller than the spinal evidence base.

These limited data are consistent with, but do not by themselves prove, a preventive rather than purely reactive approach in selected high-risk long-bone lesions. Assessment should integrate pain phenotype, anatomical location, lesion size, cortical destruction, lytic or mixed pattern, response to systemic treatment, functional demand and expected survival. The Mirels score remains a useful screening tool for impending fracture risk, particularly in appendicular lytic lesions. A score of 9 or higher generally supports prophylactic fixation, whereas a score of 8 represents a grey zone requiring specialist judgement. However, its modest specificity and limited integration of CT-defined cortical involvement, anatomical biomechanics, load-bearing demand, tumour biology and treatment response mean that it should not be used in isolation. Future fracture-risk models should integrate cross-sectional imaging, biomechanical parameters, oncological disease behaviour and treatment response. Artificial intelligence-based approaches, in particular, will require robust external validation before routine clinical use [6,16,50,51].

**Figure 4:**
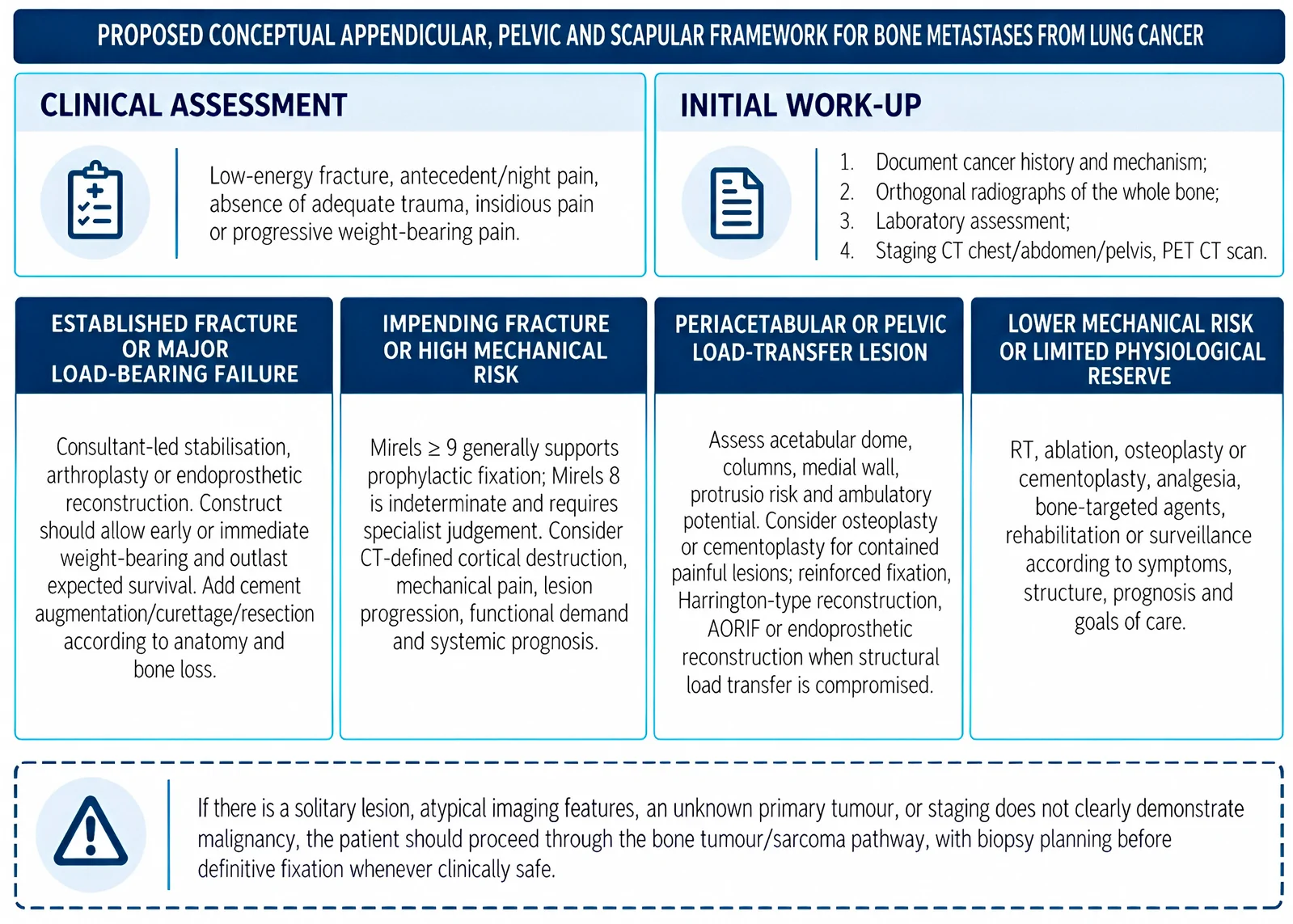
Proposed conceptual decision framework for appendicular, pelvic and scapular bone metastases from lung cancer. The framework integrates diagnostic safety, fracture-risk assessment, durable reconstruction, and multidisciplinary local treatment planning, informed by lung cancer-specific evidence and guideline-based principles. Its structure reflects author synthesis and should be regarded as conceptual rather than prescriptive [6,16,50,51].

Periacetabular and pelvic lesions require additional attention because they threaten load transfer through the hip and may compromise sitting, standing and ambulation even without a classic long-bone fracture pattern. Lee (2022) reported improved pain and ambulatory status after ablation, osteoplasty, reinforcement and internal fixation (AORIF), supporting continuation of oncological care in selected patients [91]. Solitary lesions, atypical imaging features or metastatic bone disease without a clear primary should still follow an appropriate diagnostic and biopsy pathway before definitive fixation whenever clinically safe. Incidental high-risk lesions identified on staging imaging should be approached through the same structured pathway as symptomatic disease, particularly when they involve load-bearing bone or show features suggestive of impending mechanical failure [16,50,51]. These review observations are integrated with external orthopaedic oncology guidance in the proposed appendicular, pelvic and scapular conceptual framework shown in Figure 4.

Another major finding of this review is that prognosis in metastatic lung cancer is increasingly shaped by molecular profile, treatment responsiveness and access to effective systemic therapy. Several included studies identified genotype, targeted therapy and broader systemic treatment access as key prognostic factors. This has direct orthopaedic implications: surgical planning can no longer rely exclusively on historical assumptions of uniformly short survival. In selected patients with actionable molecular alterations or favourable treatment response, prolonged survival may require constructs that remain mechanically durable over time and outlast the patient’s expected survival. Conversely, when prognosis is poor and systemic options are limited, the primary objective should be proportionate palliation. Underestimating survival may lead to mechanically insufficient constructs, converting prognostic error into orthopaedic failure. Overestimating survival may expose patients to excessive operative burden without meaningful functional gain [60,64,74,92].

Classical prognostic systems may underestimate survival in selected patients because they do not fully capture contemporary molecular stratification, targeted therapy, immunotherapy responsiveness and functional heterogeneity. Newer lung cancer-specific models incorporating visceral metastatic burden, neurological status, molecular features and treatment access appear more informative, although traditional scores should be regarded as limited rather than obsolete. Overall, the evidence supports a biology-aware, function-oriented and neurologically informed approach to patient selection, in which formal scores are used as adjuncts rather than stand-alone determinants of treatment [59,60,65,74,78]. This distinction between survival-related determinants and determinants of local orthopaedic benefit is summarised in Supplementary Table S8.

Limited survival should not automatically exclude local orthopaedic intervention when an urgent mechanical or neurological problem is otherwise likely to cause rapid loss of function. In biologically aggressive disease such as SCLC-related MSCC, observational surgical series report symptomatic and functional improvement despite short median survival. However, these data do not establish that surgery improves survival. The value of intervention in such patients should therefore be judged primarily against realistic goals of pain control, neurological preservation, functional independence and QoL, rather than life expectancy alone [6,16,50,62].

The oligometastatic subgroup requires careful restraint in interpretation. Better outcomes have been reported after multimodal radical-intent treatment, including local tumour control and prolonged survival estimates. However, these patients are biologically and clinically distinct, and their results should not be extrapolated to the wider population with metastatic lung cancer. Treatment of oligometastatic bone disease should therefore remain a patient-tailored multidisciplinary decision rather than a generalisable indication for aggressive local therapy [83].

The complication profile reported across the reviewed studies reinforces the need for careful patient selection and realistic counselling. Perioperative risk should be considered as part of the therapeutic balance between procedural burden and the expected functional or palliative benefit. Apparent differences in tolerability between open and minimally invasive approaches should be interpreted cautiously because indications and patient populations differed across studies. Importantly, complication risk should not be treated as merely a technical concern. Pulmonary reserve, nutritional status, thrombotic vulnerability, frailty and the ability to tolerate postoperative setbacks all influence whether the expected benefit of intervention remains proportionate. This is especially relevant when the anticipated gain is primarily palliative or functional rather than survival-related. In such circumstances, perioperative burden is not incidental to decision-making, it is part of the therapeutic equation itself [59,67,69,70,71,77,93]. The apparent lower physiological burden of minimally invasive treatment should not be interpreted as a direct comparative safety advantage, because the studies used different indications and selected different patient populations.

Several limitations of the current evidence base must be acknowledged. No randomised trials evaluating orthopaedic surgical or minimally invasive strategies in lung cancer bone metastases were identified. Most included studies were retrospective and many were small, single-centre cohorts. This creates a substantial risk of selection bias and confounding by indication: patients selected for surgery often had greater local mechanical or neurological threat, but they could simultaneously have better performance status, physiological reserve or expected survival than patients managed non-operatively. Conversely, non-surgical groups may include patients with more advanced systemic disease or frailty. These opposing selection mechanisms can either exaggerate or attenuate apparent treatment effects, so unadjusted differences in survival, function or complications cannot be interpreted as causal. Many studies lacked a contemporaneous control group, anatomical sites were often pooled, direct procedure comparisons were limited, adjusted effect estimates with 95% confidence intervals were inconsistently reported, and patient-reported outcome measures were variable. The evidence remains especially limited for appendicular and pelvic lesions, prophylactic intervention and direct comparison of open, minimally invasive and non-operative local strategies [9,59,77]. For future orthopaedic studies in this population, we consider at least 6 months of follow-up among survivors a pragmatic minimum for evaluating durability, reoperation and later complications, with 30- and 90-day morbidity reported separately and follow-up to death or censoring for survival outcomes; 12 months is preferable when patient survival permits. Accordingly, the present review supports broad clinical principles and structured decision-making more strongly than rigid procedure-specific algorithms. The proposed local treatment modality framework summarises how each major local treatment option may be matched to its likely indication, expected benefit and key limitation (Supplementary Table S9).

Despite these limitations, this review has important strengths. It focuses specifically on lung cancer rather than mixed-primary populations, includes axial and extraspinal disease, reflects the contemporary systemic-treatment era and is based on a broad multi-database search with a narrative synthesis appropriate to the heterogeneity of the evidence. Its main contribution is to identify recurrent lung cancer-specific clinical patterns while making clear where evidence is sparse. The proposed figures should therefore be understood as conceptual decision frameworks that integrate three distinct sources: observations from the included lung cancer-specific studies, recommendations from external guidelines and orthopaedic oncology standards, and author synthesis and expert consensus used to organise those elements into a pathway. They are not validated clinical algorithms and should not substitute for multidisciplinary judgement [61,63,72,85,88].

## 5. Conclusions

In summary, the available lung cancer-specific evidence suggests that orthopaedic care can contribute meaningfully to pain control, preservation of mechanical stability, neurological function and mobility when treatment is appropriately selected and timed. However, the evidence base is predominantly observational, is concentrated on spinal metastases and has low to very low outcome-level certainty. The central clinical question is therefore not whether a single procedure is superior, but whether a local intervention is proportionate to the patient’s neurological or mechanical threat, functional reserve, systemic disease trajectory and treatment goals.

Across the included studies, local orthopaedic interventions were most consistently associated with pain relief, preservation or recovery of ambulation, mechanical reinforcement, neurological improvement and maintenance of QoL. A direct survival benefit from surgery or other orthopaedic procedures was not established. Survival was more consistently associated with performance status, visceral disease, neurological reserve, molecular profile and access to effective systemic therapy. These findings should be interpreted as associations because treatment allocation was non-random and confounding by indication was substantial.

Decision-making should remain site-specific, multidisciplinary and proportionate. For spinal disease, the review provides the strongest support for prioritising neurological threat, epidural compression and mechanical instability. For appendicular and pelvic disease, lung cancer-specific evidence is much more limited, so fracture-risk assessment, reconstruction strategy and treatment sequencing rely to a greater extent on external orthopaedic oncology guidance and expert judgement. Open surgery and minimally invasive techniques should therefore be viewed as complementary options rather than as options in a fixed hierarchy. The figures proposed in this review are conceptual, guideline-informed decision frameworks and require prospective validation before they can be used as formal clinical algorithms.

## Figures and Tables

**Table 1:** Characteristics and main findings of spinal-predominant included studies.

Author(s)	Year	Study Type	*n*	Primary Tumour	Metastases (N)	Interventions	Reported Outcomes	Follow-Up Duration
Amelot et al. [60]	2020	Prospective cohort	210/794	NSCLC (EGFR+ ALK+)	N = 818 spinal Metastases. Median = 3 lesions/patient (range 1–17). Synchronous *n* = 122 (61%).	Surgery: posterior decompression laminectomy ± stabilisation and/or fixation, vertebroplasty or partial corpectomy. Non-surgical/adjuvant: RT, CTx, immunotherapy.	Survival; Complications; Prognostic Factors.	Until death or last contact; inclusion 2014–2017.
Chen et al. [61]	2021	Retrospective cohort	83/83	NSCLC SCLC Other.	Single vertebral body *n* = 46; Multiple *n* = 37; Extraspinal bone metastases (absent, *n* = 29; 1–2, *n* = 19; ≥3, *n* = 35).	Surgery: excisional surgery or palliative decompressive laminectomy + posterior fixation. Adjuvant: RT, CTx, targeted therapy.	Pain; Function; Survival; Prognostic Factors.	Treated 2009–2021; functional assessments at 1 week, 1 month and 6 mo.
Gao et al. [62]	2020	Retrospective cohort	30/30	SCLC	Single spinal lesion *n* = 24; multiple *n* = 6; extraspinal bone metastases *n* = 5. Synchronous *n* = 24. MSCC (All).	Surgery: circumferential decompression, tumour excision and stabilisation. Adjuvant: CTx, RT and bisphosphonates.	Pain; Function or QoL; Survival; Complications; Prognostic Factors.	Mean = 11.8 mo. (range 1–68).
Huang et al. [63]	2024	Retrospective cohort	172/172	NSCLC	Solitary/diffuse spinal lesions not quantified. Synchronous *n* = 102 (59%).	Palliative decompression. Excisional surgery: tumour curettage or debulking or total vertebrectomy. Adjuvant: targeted drugs, BMAs and RT.	Function; Survival; Complications.	Mean = 432 days.
Jung et al. [64]	2021	Retrospective cohort	58/58	NSCLC	Mean spinal metastases = 1.1–2.5 by surgical group.	Laminectomy + fusion; corpectomy + fusion; spondylectomy + fusion. Adjuvant: post-op CTx and spine RT.	Pain; Survival; Complications; Prognostic Factors.	Mean = 10.1–15.5 mo.
Kobayashi et al. [65]	2017	Retrospective cohort	10/31	NSCLC SCLC	Lung group: mean vertebral metastases: 1.4 ± 1.0; extraspinal metástases: 1.7 ± 0.5; initial stage IV *n* = 9	Surgery: posterior decompression, TES, laminectomy alone or anterior decompression/instrumentation.	Function; Complications.	Mean = 16.5 mo. (range 1–62).
Lei et al. [66]	2015	Retrospective cohort	64/64	NSCLC	Involved vertebrae: 1–2, *n* = 36; ≥3 *n* = 28; Other bone metastases, *n* = 48.	Posterior decompression and spinal stabilisation. Adjuvant: RT, CTx and gefitinib.	Function; Survival; Mortality.	Mean survivor follow-up = 9.7 mo.
Lei et al. [56]	2016a	Retrospective cohort	73/73	NSCLC SCLC	1–2 vertebrae *n* = 38; ≥3 *n* = 35; other bone metastases *n* = 57.	Surgical decompression and spinal stabilisation. Adjuvant: RT, CTx and gefitinib.	Function; Survival; Mortality; Prognostic Factors.	Mean survivor follow-up = 7.6 mo;
Lei et al. [57]	2016b	Retrospective cohort	73/73	NSCLC SCLC	1–2 vertebrae *n* = 38; ≥3 *n* = 35; other bone metastases *n* = 57.	Posterior decompression and stabilisation. Test cohort and validation cohort for prognostic scoring. Adjuvant: RT, CTx and gefitinib.	Function; Survival; Mortality; Complications.	Median survival = 6 mo; median follow-up = 3 mo.
Li et al. [67]	2015	Retrospective cohort	39/39	NSCLC stage IV	Multifocal vertebral involvement common.	PVP followed by IMRT.	Pain; QoL or function; Survival; Complications.	Assessments up to 24 mo; longest survival = 66 mo.
Li et al. [68]	2025	Retrospective cohort	128/128	NSCLC SCLC Other	Median total bone Metastases, *n* = 3; Median spinal Metastases, *n* = 2.	Palliative surgery: decompression, stabilisation or fixation and piecemeal tumour resection; posterior or anterior approach. RT and systemic treatment.	Pain; Function or QoL; Prognostic Factors.	Median = 6 mo ± 2 mo.
Lin et al. [69]	2020	Retrospective cohort	65/65	NSCLC	Single level *n* = 44; multiple levels *n* = 21; Synchronous *n* = 26; Metachronous *n* = 39.	Long posterior instrumentation ± posterior decompression. Post-op groups: EGFR-TKI vs. platinum-based CTx. Post-op RT.	Pain; Function; Survival; Complications.	2001–2011; followed until death.
Liu et al. [70]	2023	Retrospective case-control	58/58	NSCLC	Single spinal metastasis *n* = 58.	PVP alone vs. MWA + PVP.	Pain; Function or QoL; Complications; Local Progression	Median = 15–16 mo.
Park et al. [71]	2016	Prospective cohort	50/50	NSCLC	Spinal metastases: ≥3, *n* = 31; 1–2, *n* = 19; Extraspinal bone Metastases *n* = 29	Posterior corpectomy + fixation; laminectomy + posterior fixation; or anterior/lateral corpectomy + fixation. Adjuvant: post-op CTx.	Function; Survival; Complications; Prognostic Factors.	Median survival = 5.2 mo. Functional evaluation 1–2 mo post-op.
Park et al. [72]	2023	Retrospective cohort	44/44	NSCLC	Solitary and multiple involvement	Surgery + RT vs. RT alone.	Function; Survival; Fracture; Complications.	Mean = 12.3 and 11.3 mo.
Rothrock et al. [73]	2021	Retrospective cohort	309/ 1515	Mixed primary cohort.	Not specified	Separation surgery and spinal instrumentation, open or minimally invasive posterior approach. Adjuvant: conventional RT or SBRT in all patients.	Survival; Mortality.	Median = 28.9 mo.
Takahashi et al. [74]	2025	Prospective cohort	74/74	NSCLC SCLC	Not specified	Surgical decompression and spinal stabilisation.	QoL; Survival; Prognostic Factors.	Mean = 257 ± 190 days.
Tang et al. [75]	2015	Retrospective cohort	122/122	NSCLC	Single vertebra *n* = 77; multiple *n* = 39; synchronous *n* = 99. MSCC (All)	Surgery within 3 days: TES or subtotal, piecemeal resection or laminectomy. Comparisons by adjuvant RT, targeted therapy, bisphosphonates. Platinum-based CTx.	Pain; Function; Survival; Mortality; Prognostic Factors.	Median = 13.5 mo (range 3–54).
Tang et al. [76]	2016	Prospective cohort	133/133	NSCLC	Solitary, *n* = 7; Multiple, *n* = 126. Synchronous Metastases in 90%.	Surgery + adjuvant therapy vs. non-surgical adjuvant medical therapy. Surgical procedures included TES, subtotal resection, piecemeal spondylectomy and/or laminectomy with reconstruction.	QoL; Survival; Complications.	QoL at baseline, 1, 3, 6 and 9 mo; Survival until final analysis.
Truong et al. [77]	2020	Retrospective cohort	87/87	NSCLC SCLC	Not specified	Decompression, tumour resection and instrumented stabilisation: mainly corpectomy + posterior fixation. Adjuvant: RT and CTx.	Pain; Function; Survival; Complications.	Mean = 7.5 ± 2.4 mo.
Uei et al. [78]	2017	Retrospective cohort	207/207	NSCLC SCLC Other	Vertebral lesions: 1, *n* = 57; 2, *n* = 29; ≥3, *n* = 121.	Group B (≤2005): surgery or conservative treatment. Group A (2006–2016): surgery or conservative treatment with increased use of targeted drugs and BMAs.	Pain; QoL or function; Survival; Prognostic Factors.	Survival follow-up = 114 mo in Group A.
Wu et al. [79]	2024	Retrospective cohort	71/71	NSCLC	Solitary vertebral Lesions, *n* = 46; multiple lesions, *n* = 25. Total = 109 lesions. Synchronous Metastases in >30%.	CT-guided MWA followed by PVP.	Pain; Function; Complication; Local Progression.	Pre-op and 24H, 1, 4, 12 and 24 wk.
Xu et al. [80]	2021	Retrospective cohort	55/55	NSCLC	Solitary *n* = 15; double *n* = 19; multiple *n* = 21; extraspinal bone metastases *n* = 20 MSCC (All)	Massive spondylectomy with circumferential decompression and stabilisation after local RT failure.	Function; Survival; Local Control; Prognostic Factors.	Every 3 mo until death; maximum = 84 mo.
Yang et al. [81]	2017	Retrospective cohort	140/140	NSCLC	Solitary *n* = 86; Multiple *n* = 54 limited to 1–3 adjacent segments.	Conservative treatment; PVP + adjuvant therapy; open surgery + adjuvant therapy.	Pain; Function; Survival. Prognostic Factors.	Every 3 mo for 6 mo. Then, every 6 mo until death; Median survival = 29 mo.
Zhai et al. [59]	2023	Retrospective cohort	112/112	NSCLC SCLC	Multiple segments, *n* = 76; Single segment, *n* = 36; Synchronous *n* = 82.	Complete decompression (TES or debulking or separation surgery) vs. palliative surgery (decompression or PVP). Adjuvant: RT, CTx, targeted therapy and immunotherapy.	Pain; Function; Survival; Complications; Prognostic Factors.	Median = 47.4 mo (range 13–142).
Zhang et al. [36]	2016	Retrospective cohort	46/46	NSCLC	1 segment, *n* = 25; 2 segments, *n* = 9; ≥3 segments, *n* = 12.	RT alone vs. surgery + post-op RT. Surgery included wide excision/fixation or palliative laminectomy/RFA/vertebroplasty/fixation.	Pain; Function or QoL; Survival; Complications.	Mean = 8.8 mo. (range 2–25).
Zhong et al. [82]	2022	Retrospective cohort	47/47	Lung cancer (subtype not specified)	Not specified	Posterior decompression and/or fixation + intraoperative MWA vs. decompression and/or fixation alone; PMMA reinforcement for osteolytic lesions.	Pain; Function; Survival; Complications.	Median = 12 mo. (range 3–24).

**Table 2:** Characteristics and main findings of appendicular, pelvic or mixed-site included studies.

Author(s)	Year	Study Type	*n*	Primary Tumour	Metastases (N)	Interventions	Reported Outcomes	Follow-Up Duration
Cho et al. [9]	2019	Retrospective cohort	202/ 202	NSCLC SCLC	Multiple distant metastases including bone *n* = 128 (63.4%); Synchronous (All)	Surgery: curettage, IMN, plate fixation and cementing. Non-surgical: palliative care without orthopaedic surgery. Adjuvant: CTx and RT.	Function; Survival; Prognostic Factors.	Mean follow-up = 24 mo.
Congedo et al. [83]	2022	Retrospective cohort	27/27	NSCLC	Solitary, *n* = 23; two metastases, *n* = 4. Synchronous (All)	Surgery: radical resection of bone lesion. Local RT: palliative or curative intent. Systemic/adjuvant therapies varied.	Survival; Complications; Local recurrence.	Mean follow-up = 27.9 ± 20.6 mo.
Hu et al. [58]	2022	SEER (retrospective) FUSCC (prospective)	SEER 203,942 FUSCC 59	SEER: NSCLC, SCLC and others. FUSCC: NSCLC, SCLC and NOS	FUSCC: single *n* = 12, multiple *n* = 47; Synchronous metastases in 79.7%.	FUSCC: PVP; Open spinal surgery (separation surgery, TES); Limb tumour resection or prosthesis. Adjuvant: RT, CTx, EGFR-TKI.	Pain; Function; Survival.	SEER Diagnosis 2010–2017; FUSCC enrolled 2017–2020 and followed until Nov 2021.
Kim et al. [84]	2016	Prospective cohort	38/70	Mixed primary cohort.	Not specified.	Cement-augmented IMN vs conventional closed IMN. Adjuvant: RT and CTx in most augmented cases.	Pain; Survival; Complications.	Minimum = 15 mo; progression evaluated at 7.2–8.8 mo.
Wang et al. [85]	2021	Prospective cohort	746/746	NSCLC SCLC	Solitary, *n* = 267; Multiple, *n* = 319 (extent 3–4 *n* = 160).	MDT/machine-learning-guided local treatment: surgery, POP, RT; no local treatment.	Pain; QoL; Survival.	Median = 15 mo (range 6–41); assessments at 1, 6, 12 and 24 wk.
Wei et al. [86]	2015	Retrospective cohort	26/26	NSCLC SCLC	Solitary, *n* = 21; 2 lesions, *n* = 3; 3 lesions, *n* = 2.	CT-guided MWA combined with osteoplasty or cementoplasty.	Pain; Analgesia; Complications; Progression.	Median = 8.5 mo; assessments 48 h, 1 week, 1, 3 and 6 mo.

## Data Availability

No new clinical or experimental datasets were generated in this study. The data analysed in this systematic review were extracted from previously published studies identified through PubMed, Web of Science, Europe PMC, and Scopus. The complete database search strategies are provided in Appendix A, and the detailed characteristics and methodological appraisal of the included studies are provided in Supplementary Materials.

## Supplementary Materials

The following supporting information can be downloaded at: https://www.mdpi.com/article/10.3390/cancers18193228/s1, Table S1: PRISMA 2020 Checklist; Table S2: PRISMA 2020 for Abstracts Checklist; Table S3: Detailed characteristics and findings of the 33 included studies; Table S4: Item-level methodological appraisal using the JBI Critical Appraisal Checklist for Cohort Studies; Table S5: Item-level methodological appraisal using the JBI Critical Appraisal Checklist for Case-Control Studies; Table S6: Outcome-level certainty assessment of the evidence; Table S7: Proposed orthopaedic assessment framework for bone metastases from lung cancer; Table S8: Proposed determinants-of-benefit framework for orthopaedic treatment selection in bone metastases from lung cancer; Table S9: Proposed local treatment modality framework for bone metastases from lung cancer.

## Abbreviations

The following abbreviations are used in this manuscript:

ADL	Activities of Daily Living
ALK	Anaplastic Lymphoma Kinase
ARDS	Acute Respiratory Distress Syndrome
AORIF	Ablation, Osteoplasty, Reinforcement, and Internal Fixation
ASIA	American Spinal Injury Association Impairment Scale
AUC	Area Under the Curve
BI	Barthel Index
BMAs	Bone-Modifying Agents
CCI	Charlson Comorbidity Index
CI	Confidence Interval
CSF	Cerebrospinal Fluid
CT	Computed Tomography
CTx	Chemotherapy
DFS	Disease-Free Survival
DMC	Daily Morphine Consumption
EBRT	External Beam Radiation Therapy
ECOG	Eastern Cooperative Oncology Group
ECOG-PS	Eastern Cooperative Oncology Group Performance Status
EGFR	Epidermal Growth Factor Receptor
EGFR-TKI	Epidermal Growth Factor Receptor Tyrosine Kinase Inhibitor
EORTC	European Organisation for Research and Treatment of Cancer
EQ-5D	EuroQol 5-Dimension Questionnaire
EQ-5D-3L	EuroQol 5-Dimension 3-Level Questionnaire
EQ-5D-5L	EuroQol 5-Dimension 5-Level Questionnaire
ESCC	Epidural Spinal Cord Compression
FACT-G	Functional Assessment of Cancer Therapy—General
FS	Frankel Scale
FUSCC	Fudan University Shanghai Cancer Centre
HR	Hazard Ratio
ICI	Immune Checkpoint Inhibitor
IMN	Intramedullary Nailing
IMRT	Intensity-Modulated Radiotherapy
IQR	Interquartile Range
JBI	Joanna Briggs Institute
KPS	Karnofsky Performance Status
LDCT	Low-Dose Computed Tomography
LOS	Length of Stay
LTP	Local Tumour Progression
MDT	Multidisciplinary Team
MeSH	Medical Subject Headings
MRI	Magnetic Resonance Imaging
MRONJ	Medication-Related Osteonecrosis of the Jaw
mRS	Modified Rankin Scale
MSCC	Metastatic Spinal Cord Compression
MWA	Microwave Ablation
NESMS	New England Spinal Metastasis Score
NLR	Neutrophil-to-Lymphocyte Ratio
NOMS	Neurologic, Oncologic, Mechanical, Systemic Framework
NOS	Not Otherwise Specified
NRS	Numeric Rating Scale
NSCLC	Non-Small Cell Lung Cancer
ODI	Oswestry Disability Index
OR	Odds Ratio
OS	Overall Survival
PA	Psychosocial Aspects
PC	Pain Characteristics
PD-1	Programmed Cell Death Protein 1
PET	Positron Emission Tomography
PET-CT	Positron Emission Tomography–Computed Tomography
PFS	Progression-Free Survival
PMMA	Polymethylmethacrylate
POP	Percutaneous Osteoplasty
PRISMA	Preferred Reporting Items for Systematic Reviews and Meta-Analyses
PROMIS	Patient-Reported Outcomes Measurement Information System
PROSPERO	International Prospective Register of Systematic Reviews
PS	Pain Sites
PVCR	Posterior Vertebral Column Resection
PVP	Percutaneous Vertebroplasty
QLQ-BM22	Quality of Life Questionnaire–Bone Metastases Module 22
QLQ-C30	Quality of Life Questionnaire–Core 30
QoL	Quality of Life
RFA	Radiofrequency Ablation
RR	Risk Ratio
RT	Radiotherapy
SBRT	Stereotactic Body Radiation Therapy
SCLC	Small Cell Lung Cancer
SEER	Surveillance, Epidemiology, and End Results
SINS	Spinal Instability Neoplastic Score
SORG	Skeletal Oncology Research Group
SREs	Skeletal-Related Events
TES	Total En Bloc Spondylectomy
TKI	Tyrosine Kinase Inhibitor
VAS	Visual Analogue Scale
VI	Vitality Index
VTE	Venous Thromboembolism
WHO	World Health Organization

## Appendix A. Search Expressions

1. PubMed

((Lung Neoplasms OR Carcinoma, Non-Small-Cell Lung OR Carcinoma, Small Cell OR Lung cancer OR pulmonary cancer OR pulmonary neoplasm*)) AND ((Bone Neoplasms/secondary OR bone metast* OR skeletal metast* OR long bone* metast* OR spinal metast* OR vertebral metast* OR femur metast* OR humeral metast* OR tibia metast* OR Fractures, spontaneous OR pathologic fracture* OR impending fracture OR vertebral fracture*)) AND ((Orthopaedic Procedures OR orthop* OR spinal surgery OR spine surgery OR laminectomy OR corpectomy OR fracture fixation OR intramedullary nail* OR arthroplast* OR endoprosthe* OR megaprosthe* OR prosthetic replacement OR tumour prosthesis OR modular endoprosthesis OR bone plate* OR vertebroplasty OR kyphoplasty OR cementoplasty OR bone cement OR spinal fusion OR separation surgery OR bone stabilization OR spinal stabilization OR internal fixation OR screw fixation OR percutaneous fixation)).

2. Web of Science

((Lung cancer OR Pulmonary cancer OR Pulmonary neoplasm*)) AND ((bone metast* OR skeletal metast* OR long bone* metast* OR spinal metast* OR vertebral metast* OR femur metast* OR humeral metast* OR tibia metast* OR pathologic fracture* OR impending fracture OR vertebral fracture*)) AND ((orthop* OR spinal surgery OR spine surgery OR laminectomy OR corpectomy OR fracture fixation OR intramedullary nail* OR arthroplast* OR endoprosthe* OR megaprosthe* OR prosthetic replacement OR tumour prosthesis OR modular endoprosthesis OR bone plate* OR vertebroplasty OR kyphoplasty OR cementoplasty OR bone cement OR spinal fusion OR separation surgery OR bone stabilization OR spinal stabilization OR bone graft* OR internal fixation OR screw fixation OR percutaneous fixation)).

3. Europe PMC

((Lung Neoplasms OR Carcinoma, Non-Small-Cell Lung OR Carcinoma, Small Cell OR Lung cancer OR pulmonary cancer OR pulmonary neoplasm*)) AND ((bone metast* OR skeletal metast* OR long bone* metast* OR spinal metast* OR vertebral metast* OR femur metast* OR humeral metast* OR tibia metast* OR Fractures, spontaneous OR pathologic fracture* OR impending fracture OR vertebral fracture*)) AND ((Orthopaedic Procedures OR orthop* OR spinal surgery OR spine surgery OR laminectomy OR corpectomy OR fracture fixation OR intramedullary nail* OR arthroplast* OR endoprosthe* OR megaprosthe* OR prosthetic replacement OR tumour prosthesis OR modular endoprosthesis OR bone plate* OR vertebroplasty OR kyphoplasty OR cementoplasty OR bone cement OR spinal fusion OR separation surgery OR bone stabilization OR spinal stabilization OR internal fixation OR screw fixation OR percutaneous fixation)).

4. Scopus

(“Lung cancer” OR “Non-small cell Lung cancer” OR NSCLC OR “Small cell Lung cancer” OR SCLC OR “Pulmonary cancer” OR “Pulmonary neoplasm*”) AND (“bone metast*” OR “skeletal metast*” OR “spinal metast*” OR “vertebral metast*” OR “long bone metast*” OR “femur metast*” OR “humer* metast*” OR “tibia metast*” OR “pathologic fracture*” OR “impending fracture*” OR “vertebral fracture*”) AND (orthop* OR “spinal surgery” OR “spine surgery” OR laminectomy OR corpectomy OR “fracture fixation” OR “intramedullary nail*” OR arthroplast* OR endoprosthe* OR megaprosthe* OR “prosthetic replacement” OR “tumour prosthesis” OR “modular endoprosthesis” OR vertebroplasty OR kyphoplasty OR cementoplasty OR “bone cement” OR “spinal fusion” OR “separation surgery” OR “internal fixation” OR “screw fixation” OR “percutaneous fixation”).
