# Supplementary material for: Orthopaedic Management of Bone Metastases from Lung Cancer
Source: Cancers (Basel). 2026 Oct 7;18(19):3228. doi: 10.3390/cancers18193228 (PMC13664506; doi:10.3390/cancers18193228)
Supplement: Supplementary file 1 [file cancers-18-03228-s001.zip › cancers-4550473-supplementary.pdf]

Supplementary Materials

Table S1. PRISMA 2020 Checklist

Section and Topic	Item #	Checklist item	Location where item is reported
**TITLE**			
Title	1	Identify the report as a systematic review.	p. 1, Title
**ABSTRACT**			
Abstract	2	See the PRISMA 2020 for Abstracts checklist.	pp. 1–2, Abstract; Table S2
**INTRODUCTION**			
Rationale	3	Describe the rationale for the review in the context of existing knowledge.	pp. 2–3, Introduction
Objectives	4	Provide an explicit statement of the objective(s) or question(s) the review addresses.	p. 3, Introduction
**METHODS**			
Eligibility criteria	5	Specify the inclusion and exclusion criteria for the review and how studies were grouped for the syntheses.	p. 4, Materials and Methods
Information sources	6	Specify all databases, registers, websites, organisations, reference lists and other sources searched or consulted to identify studies. Specify the date when each source was last searched or consulted.	p. 4, Materials and Methods
Search strategy	7	Present the full search strategies for all databases, registers and websites, including any filters and limits used.	p. 4, Materials and Methods; pp. 25–26, Appendix A
Selection process	8	Specify the methods used to decide whether a study met the inclusion criteria of the review, including how many reviewers screened each record and each report retrieved, whether they worked independently, and if applicable, details of automation tools used in the process.	pp. 4–6, Materials and Methods and Figure 1
Data collection process	9	Specify the methods used to collect data from reports, including how many reviewers collected data from each report, whether they worked independently, any processes for obtaining or confirming data from study investigators, and if applicable, details of automation tools used in the process.	pp. 4–5, Materials and Methods
Data items	10a	List and define all outcomes for which data were sought. Specify whether all results that were compatible with each outcome domain in each study were sought (e.g. for all measures, time points, analyses), and if not, the methods used to decide which results to collect.	p. 5, Materials and Methods
	10b	List and define all other variables for which data were sought (e.g. participant and intervention characteristics, funding sources). Describe any assumptions made about any missing or unclear information.	p. 5, Materials and Methods
Study risk of bias assessment	11	Specify the methods used to assess risk of bias in the included studies, including details of the tool(s) used, how many reviewers assessed each study and whether they worked independently, and if applicable, details of automation tools used in the process.	p. 5, Materials and Methods; Tables S4–S5

Effect measures	12	Specify for each outcome the effect measure(s) (e.g. risk ratio, mean difference) used in the synthesis or presentation of results.	p. 5, Materials and Methods; Table S3 (effect estimates when reported)
Synthesis methods	13a	Describe the processes used to decide which studies were eligible for each synthesis (e.g. tabulating the study intervention characteristics and comparing against the planned groups for each synthesis (item #5)).	p. 5, Materials and Methods
	13b	Describe any methods required to prepare the data for presentation or synthesis, such as handling of missing summary statistics, or data conversions.	Not applicable (no data conversions were performed)
	13c	Describe any methods used to tabulate or visually display results of individual studies and syntheses.	p. 5, Materials and Methods; Tables 1–2 and Table S3
	13d	Describe any methods used to synthesize results and provide a rationale for the choice(s). If meta-analysis was performed, describe the model(s), method(s) to identify the presence and extent of statistical heterogeneity, and software package(s) used.	p. 5, Materials and Methods (narrative synthesis; no meta-analysis)
	13e	Describe any methods used to explore possible causes of heterogeneity among study results (e.g. subgroup analysis, meta-regression).	Not applicable (no formal subgroup analysis or meta-regression)
	13f	Describe any sensitivity analyses conducted to assess robustness of the synthesized results.	Not applicable (no sensitivity analyses)
Reporting bias assessment	14	Describe any methods used to assess risk of bias due to missing results in a synthesis (arising from reporting biases).	Not applicable (no formal reporting-bias assessment)
Certainty assessment	15	Describe any methods used to assess certainty (or confidence) in the body of evidence for an outcome.	p. 5, Materials and Methods; Table S6
**RESULTS**			
Study selection	16a	Describe the results of the search and selection process, from the number of records identified in the search to the number of studies included in the review, ideally using a flow diagram.	pp. 5–6, Materials and Methods; Figure 1
	16b	Cite studies that might appear to meet the inclusion criteria, but which were excluded, and explain why they were excluded.	p. 5, Materials and Methods; Figure 1
Study characteristics	17	Cite each included study and present its characteristics.	pp. 6–15, Results; Tables 1–2 and Table S3
Risk of bias in studies	18	Present assessments of risk of bias for each included study.	Tables S4–S5
Results of individual studies	19	For all outcomes, present, for each study: (a) summary statistics for each group (where appropriate) and (b) an effect estimate and its precision (e.g. confidence/credible interval), ideally using structured tables or plots.	pp. 6–15, Results; Tables 1–2 and Table S3

Results of syntheses	20a	For each synthesis, briefly summarise the characteristics and risk of bias among contributing studies.	pp. 6–15, Results; Tables 1–2; Tables S4–S5
	20b	Present results of all statistical syntheses conducted. If meta-analysis was done, present for each the summary estimate and its precision (e.g. confidence/credible interval) and measures of statistical heterogeneity. If comparing groups, describe the direction of the effect.	Not applicable (no statistical synthesis/meta-analysis)
	20c	Present results of all investigations of possible causes of heterogeneity among study results.	Not applicable (no formal heterogeneity analyses)
	20d	Present results of all sensitivity analyses conducted to assess the robustness of the synthesized results.	Not applicable (no sensitivity analyses)
Reporting biases	21	Present assessments of risk of bias due to missing results (arising from reporting biases) for each synthesis assessed.	Not applicable (no formal reporting-bias assessment)
Certainty of evidence	22	Present assessments of certainty (or confidence) in the body of evidence for each outcome assessed.	Table S6; pp. 15–23, Discussion and Conclusions
**DISCUSSION**			
Discussion	23a	Provide a general interpretation of the results in the context of other evidence.	pp. 15–20, Discussion
	23b	Discuss any limitations of the evidence included in the review.	p. 19, Discussion
	23c	Discuss any limitations of the review processes used.	pp. 19–20, Discussion
	23d	Discuss implications of the results for practice, policy, and future research.	pp. 16–20, Discussion
**OTHER INFORMATION**			
Registration and protocol	24a	Provide registration information for the review, including register name and registration number, or state that the review was not registered.	p. 5, Materials and Methods
	24b	Indicate where the review protocol can be accessed, or state that a protocol was not prepared.	p. 5, Materials and Methods
	24c	Describe and explain any amendments to information provided at registration or in the protocol.	Not applicable
Support	25	Describe sources of financial or non-financial support for the review, and the role of the funders or sponsors in the review.	p. 23, Funding
Competing interests	26	Declare any competing interests of review authors.	p. 23, Conflicts of Interest
Availability of data, code and other materials	27	Report which of the following are publicly available and where they can be found: template data collection forms; data extracted from included studies; data used for all analyses; analytic code; any other materials used in the review.	p. 23, Supplementary Materials and Data Availability Statement

Table S2. PRISMA 2020 for Abstracts Checklist

Section and Topic	Item #	Checklist item	Reported (Yes/No)
**TITLE**			
Title	1	Identify the report as a systematic review.	Yes
**BACKGROUND**			
Objectives	2	Provide an explicit statement of the main objective(s) or question(s) the review addresses.	Yes
**METHODS**			
Eligibility criteria	3	Specify the inclusion and exclusion criteria for the review.	Yes
Information sources	4	Specify the information sources (e.g. databases, registers) used to identify studies and the date when each was last searched.	No
Risk of bias	5	Specify the methods used to assess risk of bias in the included studies.	Yes
Synthesis of results	6	Specify the methods used to present and synthesise results.	Yes
**RESULTS**			
Included studies	7	Give the total number of included studies and participants and summarise relevant characteristics of studies.	Yes
Synthesis of results	8	Present results for main outcomes, preferably indicating the number of included studies and participants for each. If meta-analysis was done, report the summary estimate and confidence/credible interval. If comparing groups, indicate the direction of the effect (i.e. which group is favoured).	Yes
**DISCUSSION**			
Limitations of evidence	9	Provide a brief summary of the limitations of the evidence included in the review (e.g. study risk of bias, inconsistency and imprecision).	Yes
Interpretation	10	Provide a general interpretation of the results and important implications.	Yes
**OTHER**			
Funding	11	Specify the primary source of funding for the review.	No
Registration	12	Provide the register name and registration number.	No

Table S3. Detailed characteristics and findings of the 33 included studies

Author(s)	Year	Study type	n	Primary tumour	Metastases (N)	Metastatic site(s)	Interventions	Reported outcomes	Follow-up duration
Amelot et al.	2020	Prospective cohort	210/794	NSCLC<br><br>EGFR+ n=38; ALK+ n=21.	818 spinal metastases;<br><br>Median=3 lesions/patient (range 1–17);<br><br>synchronous n=122 (61%)	Axial skeleton (spine)	Surgery n=47: posterior decompression laminectomy ± stabilisation and/or fixation, vertebroplasty or partial corpectomy.<br><br>Non-surgical/adjuvant: RT, CTx, immunotherapy.	Survival: median OS=5.9 mo; no significant OS difference with surgery (p=0.141). Complications: reoperation 8.5% for infection/wound complications. Prognostic factors: longer OS with better Frankel grade, better WHO status, EGFR+ and ALK+ status.	Until death or last contact; inclusion 2014–2017.
Chen et al.	2021	Retrospective cohort	83/83	NSCLC n=71;<br><br>SCLC n=5;<br><br>Other n=7.	Single vertebral body n=46; multiple n=37;<br><br>Extraspinal bone metastases (absent n=29, 1–2 n=19, ≥3 n=35).	Axial skeleton (spine)	Surgery n=83: excisional surgery n=27 or palliative decompressive laminectomy + posterior fixation n=56.<br><br>Adjuvant: RT, CTx, targeted therapy.	Pain: VAS = 5.6±2.4 pre-op to 2.1±1.3 at 6 mo (p<0.01). Function (Frankel score): 44.6% improved ≥1 grade at 1 month. Survival: median OS 12 mo; excisional vs palliative surgery 18.8±12.4 vs 12.5±8.9 mo (p=0.0212). Prognostic/model: age ≥60, ≥3 extraspinal bone metastases and worse Frankel grade adverse; targeted therapy was incorporated into the survival nomogram; revised Tokuhashi	Treated 2009 – 2021; functional assessments at 1 week, 1 month and 6 mo.

								underestimated life expectancy.	
Cho et al.	2019	Retrospective cohort	202/202	NSCLC n=165;<br><br>SCLC n=37.	Multiple distant metastases including bone n=128 (63.4%);<br><br>Synchronous bone metastases	Appendicular skeleton	Surgery n=41: curettage, IMN, plate fixation and cementing.<br>Non-surgical n=161: palliative care without orthopaedic surgery.<br><br>Adjuvant: CTx and RT.	Function: IMN/plate fixation allowed painless full weight bearing.<br>Survival: mean survival after bone metastasis diagnosis 12.6 vs 9.1 mo for surgical vs non-surgical groups ($p<0.001$).<br>Prognostic factors: longer survival with age <60, NSCLC histology and CTx.	24 mo follow-up.
Congedo et al.	2022	Retrospective cohort	27/27	NSCLC: adenocarcinoma n=21, squamous n=5, large-cell n=1	Solitary n=23; two metastases n=4.<br><br>Synchronous bone oligometastasis	Spine n=13; long bones n=5; ribs n=4; pelvis n=2; scapula n=2; skull n=1.	Surgery n=13: radical resection of bone lesion.<br><br>Local RT n=14: palliative or curative intent.<br><br>Systemic/adjuvant therapies varied.	Survival: 1- and 5-year OS 96% and 38%; 1- and 3-year DFS 66% and 30%.<br>DFS: longer after surgical treatment; isolated RT associated with shorter DFS (HR 2.9, $p=0.03$).<br>Complications: 0% intraoperative/30-day mortality; 37% minor	Mean = 27.9±20.6 mo; median = 19 mo after last treatment.

								complications; one ARDS.<br>Local recurrence: 29.6%	
Gao et al.	2020	Retrospective cohort	30/30	SCLC	Single spinal lesion n=24; multiple n=6; extraspinal bone metastases n=5;<br><br>Synchronous Metastases in n=24.<br>MSCC (All).	Axial skeleton; cervical, thoracic and lumbar spine.	Urgent surgery within 72H: circumferential decompression, tumour excision and stabilisation.<br><br>Adjuvant: CTx, RT and bisphosphonates.	Pain: VAS 7.2 to 2.9.<br>Function/QoL: Frankel improved in 34%; self-care capacity (ECOG 0–2) 52% to 69%; EQ-5D-3L 0.3394 to 0.5884 (p<0.001).<br>Survival: median OS 9 mo; 1-year survival 29.1%.<br>Complications: surgical site infection 3.3%.<br>Prognostic factors: post-op ECOG-PS 0–2 and PD-1 immunotherapy associated with longer OS.	Mean = 11.8 mo (range 1–68).
Hu et al.	2022	Mixed:<br><br>SEER retrospective and FUSCC prospective	SEER 203,942<br><br>FUSCC 59	SEER: NSCLC, SCLC and others.<br><br>FUSCC: NSCLC n=45, SCLC n=3, NOS n=11.	FUSCC: single n=12, multiple n=47;<br><br>Synchronous metastases in 79.7%.	FUSCC:<br><br>spine (cervical, thoracic, lumbar and sacral);<br><br>femur n=2; humerus n=1	FUSCC:<br><br>PVP n=11; open spinal surgery n=45 (separation surgery n=42, TES n=3); limb tumour resection or prosthesis n=3.<br><br>Adjuvant: RT, CTx, EGFR-TKI.	Pain: NRS improved after PVP (5.0±0.9 to 0.5±0.8) and open surgery (4.8±1.3 to 1.2±1.0).<br>Function: Barthel walking and stair-climbing scores improved after open surgery.<br>Survival: median OS 11 mo.<br>Procedural: PVP had shorter operative time, blood loss and	SEER patients diagnosed 2010 – 2016/2017;<br><br>FUSCC enrolled 2017–2020 and followed until 22 Nov 2021.

								hospital stay than open surgery.	
Huang et al.	2024	Retrospective cohort	172/172	NSCLC: adenocarcinoma n=149, non-adenocarcinoma n=23.	Solitary/diffuse spinal lesions not quantified; Synchronous Metastases in n=102 (59%)	Axial skeleton: cervical to lumbosacral spine.	Palliative decompression n=89. Excisional surgery n=83: tumour curettage or debulking or total vertebrectomy. Adjuvant: targeted drugs, BMAs and RT.	Function: Frankel D/E 73% to 80% after excisional surgery and 63% to 69% after palliative surgery at 1 month. Survival: mean OS 272 days; 1- and 3-month survival 92% and 75%. Complications: reoperation 19% after excisional surgery and 6% after palliative surgery. Prognostic scores: Tomita, revised Tokuhashi and Tokuhashi lung scores showed limited predictive accuracy; the Tokuhashi lung score underestimated overall survival by approximately 35–40%.	Mean = 432 days.
Jung et al.	2021	Retrospective cohort	58/58	NSCLC	Mean spinal metastases = 1.1–2.5 by surgical group.	Axial skeleton: thoracic and lumbar spine.	Laminectomy + fusion n=21; corpectomy + fusion n=24; spondylectomy + fusion n=13. Adjuvant: post-op CTx and spine RT.	Pain: NRS improved in all groups. Function: ECOG improved/maintained in 58.6% at 3 mo. Survival: mean OS 23.3, 34.8 and 47.9 mo after laminectomy, corpectomy and spondylectomy (p=0.043).	Visits at 1, 3, 6, 9 and 12 mo, then every 3 mo; mean follow-up = 10.1–15.5 mo.

								Complications/revision: revision 19.0%, 25.0% and 7.7%, respectively. Prognostic factors: post-op CTx and more extensive surgery associated with longer survival; higher CCI and more spinal metastases adverse.	
Kim et al.	2016	Prospective cohort	38/70	Lung cancer subgroup. Other primaries also included.	Not explicitly specified.	Appendicular skeleton: femur and humerus.	Cement-augmented IMN vs conventional closed IMN. Adjuvant: RT and CTx in most augmented cases.	Pain: cement-augmented IMN provided greater pain reduction than conventional IMN, with immediate postoperative VAS 3.8 vs 6.0 and follow-up VAS 3.3 vs 6.6 (both $p<0.001$). Survival: mean 10.1 vs 9.2 mo. Complications: 9% vs 15%. Local tumour progression suppression: 50% vs 8% ($p=0.022$).	Minimum = 15 mo; progression evaluated at mean = 7.2–8.8 mo.
Kobayashi et al.	2017	Retrospective cohort	10/31	Lung cancer: NSCLC n=9, SCLC n=1	Lung group: mean vertebral metastases 1.4±1.0; extraspinal metastases 1.7±0.5; initial stage IV n=9	Axial skeleton (spine)	Surgery: posterior decompression, TES, laminectomy alone or anterior decompression/instrumentation.	Function: Frankel grade improved in the lung cancer group, mainly to Frankel D ($p=0.01$). Survival: median 7.5 mo. Complications: one death from surgical site infection at 1 month.	Mean = 16.5 mo (range 1–62).

								Prognostic score: Revised Tokuhashi score low (4.8±1.6) and underestimated survival in some patients.	
Lei et al.	2015	Retrospective cohort	64/64	NSCLC	1–2 involved vertebrae n=36; ≥3 n=28; other bone metastases n=48.	Axial skeleton (thoracolumbar MSCC)	Posterior decompression and spinal stabilisation n=64.<br><br>Adjuvant: RT, CTx and gefitinib.	Function: 69% ambulatory at 4 wk; 51.6% of non-ambulatory patients regained walking. Survival: median OS 6.3 mo; 6- and 12-month survival 52.6% and 23%. Mortality: 4-week surgical mortality 9.4%. Prognostic factors: ECOG-PS 3–4, ≥3 vertebrae, visceral metastases and motor decline <14 days adverse.	Mean survivor follow-up = 9.7 mo.
Lei et al.	2016 a	Retrospective cohort	73/73	Lung cancer: adenocarcinoma n=52; non-adenocarcinoma n=21, including SCLC n=9.	1–2 vertebrae n=38; ≥3 n=35; other bone metastases n=57.	Axial skeleton (spine MSCC)	Surgical decompression and spinal stabilisation.<br><br>Adjuvant: RT, CTx and gefitinib.	Function: 68.5% ambulatory at 4 wk; 51.5% of pre-op Frankel B/C patients regained walking. Survival: median OS 6.0 mo; 6- and 12-month survival 49% and 22%. Mortality: 4-week post-op = 11%. Prognostic factors: preoperative ambulation, targeted	Mean survivor follow-up = 7.6 mo; function assessed at 4 wk.

								therapy, 1–2 vertebrae, no visceral metastases and motor decline >14 days associated with longer OS.	
Lei et al.	2016 b	Retrospective cohort	73/73	Lung cancer: adenocarcinoma n=52; non-adenocarcinoma n=21, including SCLC n=9	1–2 vertebrae n=38; ≥3 n=35; other bone metastases n=57	Axial skeleton; spine MSCC	Posterior decompression and stabilisation. Test cohort n=37 and validation cohort n=36 for prognostic scoring. Adjuvant: RT, CTx and gefitinib.	Function: 68.5% ambulatory at 4 wk; 51.5% of non-ambulatory patients regained walking. Survival: median OS 6.2 mo in test group and 6.0 mo in validation group; favourable scores had OS 10.7–10.8 vs 2.6–2.7 mo. Mortality/complications: 4-week post-op mortality 11%; 2 patients lost ambulation due to wound infection.	Median survival = 6 mo; median follow-up among survivors = 3 mo.
Li et al.	2015	Retrospective cohort	39/39	NSCLC stage IV	Multifocal vertebral involvement common	Axial skeleton; mainly T10 – L5; cervical and sacral levels also included	PVP followed by IMRT about 3 days later.	Pain: VAS 7.93±1.09 to 2.59±1.55 at 12 mo ($p<0.05$). QoL/function: ADL 50.9±11.7 to 82.3±9.9; walking, transfer and stair scores improved. Survival: median OS 13 mo. Complications: cement leakage 7.7%.	Assessments up to 24 mo; longest survival = 66 mo.
Li et al.	2025	Retrospective cohort	128/128	NSCLC 85.7%; SCLC 7.1%;	Median total bone metastases = 3;	Axial skeleton	Palliative surgery: decompression, stabilisation or fixation and piecemeal tumour resection; posterior or anterior approach.	Pain: median VAS = 7 (pre-op) to 0 at 6mo. Function/QoL: non-ambulatory patients	6 mo ±2 mo.

				Other 7.1%.	median spinal metastases = 2.		RT and systemic treatment.	28.1% to 10.2%; median KPS 60 to 90 at 6 mo. Prognostic factors: male sex associated with short-term improvement; more bone metastatic sites predicted less functional improvement.	
Lin et al.	2020	Retrospective cohort	65/65	NSCLC	Single level n=44; multiple levels n=21; synchronous n=26; metachronous n=39.	Axial skeleton (thoracolumbar spine)	Long posterior instrumentation ± posterior decompression. Post-op groups: EGFR-TKI n=35 vs platinum-based CTx n=30. Post-op RT.	Pain: VAS 6.8±0.5 to 3.8±1.3 at discharge. Function: Frankel improved ≥1 grade in 67% with pre-op deficits; ambulation in 95.4%. Survival: median post-op survival 7.0 mo; no difference EGFR-TKI vs CTx (p=0.61). Complications: superficial wound infection, wound dehiscence after RT and pyogenic vertebral osteomyelitis.	2001–2011; followed until death.
Liu et al.	2023	Retrospective case-control	58/58	NSCLC	Single spinal metastasis n=58.	Axial skeleton: (thoracic n=11, lumbar n=47)	PVP alone n=30 vs MWA + PVP n=28.	Pain: similar up to 12 wk; at 24 wk MWA+PVP had lower VAS and DMC (p=0.03 and p=0.01). Function/QoL: at 24 wk MWA+PVP had lower ODI and higher	Median = 15–16 mo; assessments at 1, 4, 12 and 24 wk; imaging every 3 mo.

								KPS/sleep scores. Complications: cement leakage 7% vs 40% (p=0.03). Local progression: 7.14% vs 33.0% (p=0.02); MWA+PVP was an independent protective factor on multivariate analysis (p=0.028).	
Park et al.	2016	Prospective cohort	50/50	NSCLC	≥3 spinal metastases n=31;<br>1–2 n=19;<br><br>extraspinal bone metastases n=29	Axial skeleton (thoracic, lumbar and cervical spine)	Posterior corpectomy + fixation, laminectomy + posterior fixation, or anterior/lateral corpectomy + fixation.<br><br>Adjuvant: post-op CTx.	Function: 59.0% of previously non-ambulatory patients regained walking and 66.0% improved by at least one ECOG grade. Survival: median 5.2 mo; 3-, 6- and 12-month survival 66.0%, 49.4% and 22.4%. Complications: major complications 34%; 30-day mortality 10%. Prognostic factors: surgery within 72 h of deficit associated with post-op ambulation and longer survival.	Median survival = 5.2 mo; functional evaluation 1–2 mo post-op or bedside if shorter survival.
Park et al.	2023	Retrospective cohort	44/44	NSCLC	Solitary and multiple involvement	Axial skeleton	Surgery + RT n=22 vs RT alone n=22.	Function: neurological deterioration 22.7% in RT-alone; Frankel improvement 22.7% in surgery+RT; 66.7% of non-ambulatory surgical patients regained walking.	Assessments every 4–6 wk; mean follow-up = 12.3 and 11.3 mo.

								Survival: median OS 19 vs 8 mo (p=0.790). Fracture: delayed fractures occurred in RT-alone group; surgery prevented fracture progression in potentially unstable spines. Complications: wound infection 4.5%.	
Rothrock et al.	2021	Retrospective cohort	309/1515	Lung cancer subgroup within mixed-primary cohort.	Not specified	Axial skeleton	Separation surgery and spinal instrumentation, open or minimally invasive posterior approach.<br><br>Adjuvant: conventional RT or SBRT in all patients.	Survival: median OS 4.6 mo; 6-month, 1-year and 2-year survival 40.8%, 23.8% and 14.7%. Mortality: 30-day 9.1%; 90-day 36.6%. Temporal trend: survival improved by 2% per calendar year of surgery (p=0.03).	Median survivor follow-up = 28.9 mo; lung-specific follow-up not reported.
Takahashi et al.	2025	Prospective cohort	74/74	NSCLC n=69;<br><br>SCLC n=5.	Not specified	Axial skeleton	Surgical decompression and spinal stabilisation.	QoL: EQ-5D-5L mobility, usual activities and pain/discomfort improved post-op. Survival: mean post-op survival 343 days; 6-month and 1-year survival 73.1% and 56.3%. Prognostic factors: targeted therapy, higher mobility score and higher	Mean = 257 ± 190 days.

								Vitality Index associated with longer survival.	
Tang et al.	2015	Retrospective cohort	122/122	NSCLC; adenocarcinoma n=88, squamous n=28; all MSCC	Single vertebra n=77; multiple n=39; synchronous n=99	Axial skeleton; thoracic, lumbar and cervical spine	Surgery n=116 within 3 days: TES n=17 or subtotal, piecemeal resection or laminectomy n=99. Comparisons by adjuvant RT, targeted therapy, bisphosphonates. Platinum-based CTx.	Pain: VAS 7.76±1.06 to 1.97±1.04. Function: 67.2% Frankel E post-op; 38.8% of Frankel A–C regained walking. Survival: median OS 12 mo; 1-, 2-, 3-year survival 58.5%, 27.6%, 9.3%. Mortality: perioperative 5.0%. Prognostic factors: post-op Frankel E, adjuvant RT, targeted therapy and ECOG-PS 1–2 associated with longer OS.	Median = 13.5 mo (range 3–54).
Tang et al.	2016	Prospective cohort	133/133	NSCLC	Solitary n=7; multiple n=126;<br><br>Synchronous Metastases in 90%.	Axial skeleton	Surgery + adjuvant therapy n=68 vs non-surgical adjuvant medical therapy n=65. Surgical procedures included TES, subtotal resection, piecemeal spondylectomy and/or laminectomy with reconstruction.	QoL: FACT-G total and domains higher after surgery than baseline and non-surgical group (p<0.001). Survival: surgery group median survival 14 vs 13 mo; 1-, 2-, 3-year survival 66.0%, 39.0%, 17.4% vs 52.5%, 17.0%, 6.8% (p=0.020). Complications: surgical site infection 7.4%.	QoL at baseline, 1, 3, 6 and 9 mo; survival until final analysis.

Truong et al.	2020	Retrospective cohort	87/87	NSCLC n=78; SCLC n=7; Neuro endocrine n=2.	Not specified	Axial skeleton (spine)	Decompression, tumour resection and instrumented stabilisation: mainly corpectomy + posterior fixation. Adjuvant: RT and CTx.	Pain: back pain improved in 78.2%. Function: walking improved in 43.7% and was maintained in 51.7%; 95.4% of non-ambulatory patients regained walking. Survival: mean 4.1±0.8 mo; 6- and 12-month survival 32.2% and 16.1%. Complications: medical 13.8%, surgical 5.7%, infection 2.3%; 30-day mortality 4.6%.	Mean = 7.5±2.4 mo.
Uei et al.	2017	Retrospective cohort	207/207	NSCLC n=167; SCLC n=30; Other n=10.	1 vertebral lesion n=57; 2 n=29; ≥3 n=121.	Axial skeleton (thoracic, lumbosacral and cervical spine)	Group B (≤2005) n=54: surgery or conservative treatment. Group A (2006–2016) n=153: surgery or conservative treatment with increased use of targeted drugs and BMAs.	Pain: improved in 92.2% and 92.6% in Groups A and B. QoL/function: BI improved in Group A (60.7 to 70.2). Survival: post-treatment survival longer in Group A (9.3±13.1 vs 5.1±5.3 mo; p<0.01). Prognostic score: Tokuhashi decreased from 85.2% to 62.1%.	Survival followed over full post-treatment course; range to 114 mo in Group A.
Wang et al.	2021	Prospective cohort	746/746	NSCLC n=524; SCLC n=32; Poorly differentiated n=190.	Solitary n=267; multiple n=319 (extent 3–4 n=160).	Spine; pelvis; femur; humerus.	MDT/machine-learning-guided local treatment: surgery n=54, POP n=104, RT n=71; no local treatment n=517.	Pain: surgery, POP and RT produced significant pain reduction, without significant between-modality differences. QoL: QLQ-BM22 scores improved after	Median = 15 mo (range 6–41); assessments at 1, 6, 12 and 24 wk.

								local treatment. Survival: median OS 18.03±0.45 mo; 1-year survival 65.55%; no significant survival difference between treatment groups. Model: decision-tree AUC values up to 0.92; accuracy 88.8% vs MDT decisions.	
Wei et al.	2015	Retrospective cohort	26/26	NSCLC n=23; SCLC n=2 carcinoid n=1.	Solitary n=21; 2 lesions n=3; 3 lesions n=2.	Pelvis and appendicular skeleton: ilium, acetabulum, ischium, femur, clavicle, sacrum, tibia.	CT-guided MWA combined with osteoplasty or cementoplasty.	Pain: VAS 7.4±1.6 to 1.2±0.8 at 6 mo ($p<0.001$). Analgesia: daily morphine 47.7±30.1 mg to 9.2±12.3 mg at 6 mo. Complications: overall complication rate 28%; no peri-procedural deaths. Progression: new painful bone metastases 7.7% at 5 mo.	Median = 8.5 mo; assessments 48 h, 1 week, 1, 3 and 6 mo.
Wu et al.	2024	Retrospective cohort	71/71	NSCLC	Solitary vertebral lesions n=46; multiple n=25. Total 109 lesions. Synchronous Metastases in >30%	Axial skeleton (thoracic and lumbar spine)	CT-guided MWA followed by PVP.	Pain: VAS 6.6±1.8 to 1.3±1.1 at 24 wk; morphine 137.2±38.7 mg to 24.8±21.0 mg ($p<0.001$). Function: ODI 32.2±8.2 to 10.6±7.6 at 24 wk. Complications: asymptomatic cement leakage 46.8%; grade 3 neural injury 1.4%; mild skin infection 0.9%.	Pre-op and 24H, 1, 4, 12 and 24 wk.

								Local progression: none during 6 mo.	
Xu et al.	2021	Retrospective cohort	55/55	NSCLC: adenocarcinoma n=42, squamous n=13.<br><br>All MSCC after RT failure	Solitary n=15; double n=19; multiple n=21; extraspinal bone metastases n=20	Axial skeleton (thoracic, cervical and lumbar spine)	Massive spondylectomy with circumferential decompression and stabilisation after local RT failure.	Function: neurological improvement 83.6%; improvement from FS C to FS E in 12 patients.<br>Survival: mean 14.98±14.81 mo; median 10 mo.<br>Local control: no surgical-site recurrence.<br>Prognostic factors: pre-op FS A/B, visceral metastases, D-dimer ≥1000 mg/L and neutrophil-to-lymphocyte ratio ≥5 associated with shorter survival.	Every 3 mo until death; maximum about 84 mo.
Yang et al.	2017	Retrospective cohort	140/140	NSCLC: squamous n=75, adenocarcinoma n=65.	Solitary n=86; multiple n=54 limited to 1–3 adjacent segments.	Axial skeleton (cervical, thoracic, lumbar and sacral spine)	Conservative treatment n=47; PVP + adjuvant therapy n=44; open surgery + adjuvant therapy n=49.	Pain: VAS improved in PVP and open surgery groups.<br>Function: ASIA E at last follow-up in 14.3%, 6.8% and 4.3% of surgery, PVP and conservative groups.<br>Survival: median OS 13 mo with open surgery and 7 mo with conservative/PVP groups ($p<0.01$).<br>Prognostic factors: ECOG-PS 1–2, better ASIA grade, adjuvant RT, targeted therapy	Every 3 mo for 6 mo, then every 6 mo until death; surgical survivors median 29 mo.

								and bisphosphonates associated with longer survival.	
Zhai et al.	2023	Retrospective cohort	112/112	NSCLC n=103;<br><br>SCLC n=8;<br><br>Carcinoid n=1.	Multiple segments n=76; single segment n=36;<br><br>Synchronous in n=82.	Axial skeleton (mainly thoracic spine)	Complete decompression n=59 (TES or debulking or separation surgery) vs palliative surgery n=53 (decompression or PVP).<br><br>Adjuvant: RT, CTx, targeted therapy and immunotherapy.	Pain: 68.7% pain-free post-op. Function: neurological status improved or remained normal in 83.0%; KPS improved in 83.9% with baseline KPS 10–40; complete decompression was associated with better postoperative neurological and functional outcomes than palliative surgery. Survival: median OS 16 mo; 1-, 2- and 3-year survival 63.4%, 63.4% and 22.6%. Complications: 22.3%, including pulmonary complications among severe events; neurological deterioration 5.4%. Prognostic factors: postoperative molecular targeted therapy was the only independent predictor of longer survival on multivariate analysis.	Median = 47.4 mo (range 13 – 142).
Zhang et al.	2016	Retrospective cohort	46/46	NSCLC: adenocarcinoma n=46.	1 segment n=25; 2	Axial skeleton (spine)	RT alone n=25 vs surgery + post-op RT n=21.	Pain: both groups improved; 3-month VAS lower with	Mean = 8.8 mo

					segments n=9; ≥3 segments n=12.		Surgery included wide excision/fixation or palliative laminectomy/RFA/vertebroplasty/fixation.	surgery (3.01±0.79 vs 4.34±1.47; p=0.001). Function/QoL: neurological recovery, KPS and EORTC QLQ-C30 improved more after surgery. Survival: median OS 8.4 vs 7.8 mo (p=0.24). Complications: CSF leak 4.8%, infection 9.5% after surgery; paraplegia 8.0% after RT.	(range 2–25).
Zhong et al.	2022	Retrospective cohort	47/47	Lung cancer (subtype not specified)	Not specified	Axial skeleton (thoracolumbar spine)	Posterior decompression and/or fixation + intraoperative MWA n=28 vs decompression and/or fixation alone n=19; PMMA reinforcement for osteolytic lesions.	Pain: post-op pain lower with MWA at 48 h and 1 month; no difference at 3 mo. Function: neurological improvement 80.9%; no between-group difference. Survival: median OS 14 mo; no between-group difference. Complications: CSF leak 3.6% in MWA group.	Median = 12 mo (range 3–24).

Table S4. Item-level methodological appraisal using the JBI Critical Appraisal Checklist for Cohort Studies

Study	Q1	Q2	Q3	Q4	Q5	Q6	Q7	Q8	Q9	Q10	Q11
Amelot et al. (2020)	NA	NA	Yes	Yes	Yes	Yes	Yes	UC	No	No	Yes
Chen et al. (2021)	NA	NA	Yes	Yes	Yes	Yes	Yes	Yes	No	No	Yes
Cho et al. (2019)	No	Yes	Yes	Yes	Yes	Yes	Yes	Yes	No	No	Yes
Congedo et al. (2022)	No	Yes	Yes	Yes	Yes	Yes	Yes	Yes	Yes	NA	UC
Gao et al. (2020)	NA	NA	Yes	Yes	Yes	Yes	Yes	Yes	Yes	UC	Yes
Hu et al. (2022)	Yes	Yes	Yes	Yes	Yes	No	Yes	Yes	UC	No	Yes
Huang et al. (2024)	No	Yes	Yes	Yes	Yes	NA	Yes	Yes	UC	Yes	Yes
Jung et al. (2021)	No	Yes	Yes	Yes	Yes	Yes	Yes	Yes	UC	Yes	Yes
Kim et al. (2016)	Yes	Yes	Yes	Yes	No	Yes	Yes	Yes	No	No	Yes
Kobayashi et al. (2017)	Yes	Yes	Yes	Yes	No	Yes	Yes	Yes	Yes	NA	Yes
Lei et al. (2015)	NA	NA	Yes	Yes	Yes	Yes	Yes	Yes	Yes	NA	Yes
Lei et al. (2016a)	NA	NA	Yes	Yes	Yes	Yes	Yes	Yes	Yes	NA	Yes
Lei et al. (2016b)	NA	NA	Yes	Yes	Yes	Yes	Yes	Yes	Yes	NA	Yes
Li et al. (2025)	NA	NA	Yes	Yes	Yes	NA	Yes	Yes	Yes	No	Yes
Li et al. (2015)	NA	NA	Yes	Yes	Yes	Yes	Yes	Yes	No	No	Yes
Lin et al. (2020)	Yes	Yes	Yes	Yes	Yes	Yes	Yes	Yes	Yes	NA	Yes
Park et al. (2016)	NA	NA	Yes	Yes	Yes	Yes	Yes	Yes	Yes	NA	Yes
Park et al. (2023)	No	Yes	Yes	Yes	Yes	Yes	Yes	Yes	Yes	NA	Yes
Rothrock et al. (2021)	No	Yes	Yes	Yes	No	Yes	Yes	Yes	Yes	Yes	Yes
Takahashi et al. (2025)	NA	NA	Yes	Yes	Yes	Yes	Yes	Yes	Yes	NA	Yes
Tang et al. (2015)	NA	NA	Yes	Yes	Yes	Yes	Yes	Yes	No	No	Yes
Tang et al. (2016)	Yes	Yes	Yes	Yes	No	Yes	Yes	Yes	Yes	No	Yes
Truong et al. (2020)	NA	NA	Yes	Yes	Yes	Yes	Yes	Yes	UC	No	Yes
Uei et al. (2017)	No	Yes	Yes	Yes	No	Yes	Yes	Yes	No	No	Yes
Wang et al. (2021)	No	Yes	Yes	Yes	No	Yes	Yes	Yes	UC	No	Yes
Wei et al. (2015)	NA	NA	Yes	Yes	No	Yes	Yes	Yes	UC	No	Yes
Wu et al. (2024)	NA	NA	Yes	Yes	Yes	Yes	Yes	Yes	UC	No	Yes
Xu et al. (2021)	NA	NA	Yes	Yes	Yes	Yes	Yes	Yes	Yes	NA	Yes
Yang et al. (2017)	No	Yes	Yes	Yes	No	Yes	Yes	Yes	Yes	UC	Yes
Zhai et al. (2023)	NA	NA	Yes	Yes	Yes	Yes	Yes	Yes	No	No	Yes
Zhang et al. (2016)	No	Yes	Yes	Yes	No	Yes	Yes	Yes	No	No	Yes
Zhong et al. (2022)	Yes	Yes	Yes	Yes	No	Yes	Yes	Yes	Yes	Na	Yes

Table S5. Item-level methodological appraisal using the JBI Critical Appraisal Checklist for Case-Control Studies

Study	Q1	Q2	Q3	Q4	Q5	Q6	Q7	Q8	Q9	Q10
Liu et al. (2023)	Yes	No	Yes	Yes	Yes	Yes	Yes	Yes	Yes	UC

Table S6. GRADE evidence profile by outcome domain.

Framework: Intervention outcomes were rated using the conventional GRADE approach for non-randomised intervention evidence (starting at Low certainty). The prognostic associations row was rated using GRADE guidance for prognostic factors, for which observational evidence starts at High certainty. JBI item-level assessments informed the GRADE risk-of-bias judgements; no numerical JBI score was used. Because quantitative pooling was not feasible, certainty was rated for the narrative body of evidence.

Outcome	Number of studies (N) / participants (n)	Risk of bias	Inconsistency	Indirectness	Imprecision	Certainty
**Pain relief**	**N = 19** (3 prospective cohorts, 15 retrospective cohorts, 1 case-control); **n = 2,162**	Serious[1]	Not serious[2]	Serious[3]	Not serious[4]	**VERY LOW**
**Functional / neurological recovery**	**N = 24** (2 prospective cohorts, 21 retrospective cohorts, 1 case-control); **n = 2,022**	Serious[1]	Not serious[2]	Serious[3]	Not serious[4]	**VERY LOW**
**Quality of life**	**N = 8** (3 prospective cohorts, 4 retrospective cohorts, 1 case-control); **n = 1,197**	Serious[6]	Not serious[2]	Serious[7]	Not serious[4]	**VERY LOW**
**Local tumour control / progression**	**N = 6** (1 prospective cohort, 4 retrospective cohorts, 1 case-control); **n = 307**	Serious[8]	Serious[9]	Serious[10]	Serious[11]	**VERY LOW**
**Comparative survival effect**	**N = 12** (4 prospective cohorts, 8 retrospective cohorts); **n = 1,774**	Very serious[12]	Serious[13]	Serious[14]	Serious[15]	**VERY LOW**
**Complications / procedural safety**	**N = 23** (4 prospective cohorts, 18 retrospective cohorts, 1 case-control); **n = 1,891**	Serious[16]	Serious[17]	Serious[18]	Serious[19]	**VERY LOW**
**Prognostic associations†**	**N = 18** (4 prospective cohorts, 14 retrospective cohorts); **n = 2,536**	Serious[20]	Not serious[21]	Serious[22]	Not serious[23]	**LOW**

GRADE judgements and counting rules:

Participant totals represent lung-cancer participants enrolled in studies contributing to each outcome and may exceed the number evaluable at every follow-up. The *two Lei et al.* (2016a/2016b) reports from the same 73-patient cohort were counted once within each relevant outcome. For mixed-primary studies, only the identifiable lung-cancer subgroup was counted. For *Hu et al.*, only the FUSCC clinical cohort (n=59) was counted.

1. Serious risk of bias: pain and functional estimates were dominated by uncontrolled pre/post cohorts or non-randomised comparisons. The JBI appraisal showed recurrent limitations in group comparability, control of confounding in comparative cohorts, and completeness/management of follow-up (particularly Q1, Q5 and Q9–Q10), although outcome ascertainment and statistical analysis were generally acceptable.

2. No downgrade for inconsistency: despite heterogeneous instruments, procedures and follow-up schedules, the direction of pain, functional and QoL change was broadly concordant across contributing studies.

3. Serious indirectness: the review question spans spinal, appendicular and pelvic disease, but pain and functional evidence was dominated by spinal metastases/MSCC, with substantially less extraspinal evidence.

4. No additional downgrade for imprecision: the cumulative information size was substantial and the direction of change was repeatedly observed. Confidence intervals were often absent, limiting precision of specific effect sizes, but this was not judged sufficient for a separate domain-level downgrade for these broad outcomes.

5. No upgrading: apparent large effects were predominantly derived from uncontrolled or non-randomised estimates and therefore did not meet GRADE upgrading criteria. Dose-response evidence was not demonstrated. Publication bias could not be reliably assessed because of clinical heterogeneity and the small number of studies within individual treatment contrasts.

6. Serious QoL risk of bias: non-random treatment allocation, survivorship at later assessments and incomplete follow-up could influence patient-reported outcomes. This is consistent with recurrent JBI follow-up limitations in several contributing cohorts.

7. Serious QoL indirectness: QoL evidence remained predominantly spinal and used different constructs/instruments (including FACT-G, EORTC QLQ-C30, EQ-5D, QLQ-BM22 and ODI/ADL-related measures), limiting a single review-wide inference across skeletal sites.

8. Serious local-control risk of bias: groups were non-randomised, surveillance schedules and definitions were not uniform, and several estimates came from highly selected cohorts or mixed-primary populations.

9. Serious local-control inconsistency: progression/control was variably defined as imaging progression, recurrence, revision for progression or absence of surgical-site recurrence; reported rates differed materially across modalities and anatomical settings.

10. Serious local-control indirectness: only six studies contributed and the evidence was modality- and site-specific (ablation/vertebroplasty, cement-augmented long-bone fixation, spinal surgery and oligometastatic resection).

11. Serious local-control imprecision: only 307 participants contributed, event counts were small and confidence intervals for treatment contrasts were generally unavailable.

12. Very serious survival risk of bias: all treatment comparisons were non-randomised and strongly vulnerable to confounding by indication, baseline prognosis, systemic disease burden, treatment era and access to effective systemic therapy. JBI appraisal identified imperfect group comparability and incomplete confounding control in several key comparative cohorts.
13. Serious survival inconsistency: some cohorts reported longer survival with surgery or more extensive local treatment, whereas others reported no independent survival advantage.
14. Serious survival indirectness: interventions and comparator packages differed substantially, and selected oligometastatic or surgically fit populations were not representative of the full metastatic lung-cancer population.
15. Serious survival imprecision: no pooled adjusted effect estimate was available, several direct comparisons were modest in size, and adjusted confidence intervals were inconsistently reported, leaving the magnitude of any treatment effect uncertain.
16. Serious safety risk of bias: adverse-event ascertainment was commonly secondary, definitions and follow-up windows varied, and treatment selection created baseline-risk differences. JBI follow-up limitations were frequent among contributing studies.
17. Serious safety inconsistency: studies used different complication definitions and denominators, and the event profile varied substantially by procedure type and reporting window.
18. Serious safety indirectness: open surgery, vertebral augmentation, ablation/cement procedures and appendicular fixation were used for different indications and populations, limiting a single comparative safety inference across procedures.
19. Serious safety imprecision: clinically important adverse events were often uncommon, per-procedure denominators were small and direct comparative estimates with confidence intervals were rare.
20. Serious prognostic risk of bias: many cohorts were retrospective and surgically selected; predictor selection, adjustment sets and model validation varied. The JBI appraisal generally showed better confounder identification/management than in treatment-comparison questions, supporting a one-level rather than two-level downgrade.
21. No downgrade for prognostic inconsistency: the main prognostic domains recurred across studies—better performance/neurological status and lower systemic disease burden were favourable, while actionable molecular biology and access to effective systemic therapy were repeatedly associated with longer survival.
22. Serious prognostic indirectness: most evidence came from surgically selected spinal cohorts; appendicular/pelvic and non-operative populations were under-represented, and several reported predictors were postoperative rather than pre-treatment.
23. No domain-level downgrade for prognostic imprecision: the cumulative sample was substantial and the principal prognostic domains were replicated across cohorts, although precision of individual predictor estimates and individual prediction models remains variable.

† Prognostic-specific GRADE note: For prognostic-factor questions, GRADE guidance starts a body of observational evidence at High certainty. This row is an outcome-domain appraisal of the prognostic evidence in the review and should not be interpreted as a certainty rating for any single prognostic factor or prediction model.

Table S7. Proposed orthopaedic assessment framework for bone metastases from lung cancer. Four-domain framework integrating neurological threat, mechanical risk, biological prognosis and functional reserve to guide initial orthopaedic assessment.

Based on the findings of this systematic review and informed, where applicable, by BOA/BOOS, NICE NG234 and ESMO guidance and related orthopaedic oncology frameworks [49–52]. The figure is a conceptual, guideline-informed framework; its sequencing reflects author synthesis and it has not been prospectively validated as a prescriptive algorithm.

ORTHOPAEDIC ASSESSMENT FRAMEWORK FOR BONE METASTASES FROM LUNG CANCER

PATIENT WITH SYMPTOMATIC OR HIGH-RISK BONE METASTASIS FROM LUNG CANCER

Pathway activation triggers: Low-energy fracture in a non-osteoporotic context; insidious progressive pain; night pain; mechanical weight-bearing pain; new spinal or radicular pain; motor, sensory, gait or sphincter change; incidental high-risk lesion on staging imaging.

Before definitive local treatment, confirm: Diagnostic certainty; staging status; lesion type; solitary or oligometastatic disease; unknown primary; biopsy; referral requirement; multidisciplinary decision-patient-centred goals; expected functional benefit.

WHAT PREVENTABLE HARM IS MOST LIKELY TO DETERMINE OUTCOME?

1. NEUROLOGICAL THREAT

Deficit; MSCC; ESCC grade; sphincters; ambulation; radicular pain; urgency of whole-spine MRI and MSCC pathway.

Immediate implication: Suspected neurological MSCC requires emergency pathway activation and early spine/radiation oncology input.

2. MECHANICAL RISK

SINS; Mirels; CT-defined cortical destruction; vertebral collapse; acetabular or pelvic load transfer; established fracture.

Immediate implication: Gross instability or impending fracture requires mechanical management. RT alone is insufficient for structural failure.

3. BIOLOGICAL PROGNOSIS

NSCLC/SCLC; visceral burden; molecular profile; actionable targets; systemic options; treatment response; expected survival.

Immediate implication: Procedure intensity and construct durability should reflect contemporary biology-adjusted prognosis.

4. FUNCTIONAL RESERVE AND GOALS

ECOG/KPS; frailty; ADL; pain burden; pulmonary reserve; VTE/anticoagulation; rehabilitation potential; patient priorities.

Immediate implication: Selection of the least morbid intervention capable of restoring comfort, mobilisation, dignity and treatment eligibility.

MULTIDISCIPLINARY TREATMENT SELECTION:
Open surgery or minimally invasive, RT, systemic treatment, surveillance or a combined strategy according to the dominant clinical indication and the patient's goals.

Table S8. Proposed determinants-of-benefit framework for orthopaedic treatment selection in bone metastases from lung cancer. Framework distinguishing survival-related factors from determinants of local orthopaedic benefit, including pain relief, neurological recovery, mechanical stability, ambulation and functional preservation.

Based on the findings of this systematic review and informed, where applicable, by BOA/BOOS, NICE NG234 and ESMO guidance and related orthopaedic oncology frameworks [49–52]. The figure is a conceptual, guideline-informed framework; its sequencing reflects author synthesis and it has not been prospectively validated as a prescriptive algorithm.

DETERMINANTS-OF-BENEFIT FRAMEWORK FOR ORTHOPAEDIC TREATMENT SELECTION IN BONE METASTASES FROM LUNG CANCER

Decision axis	More durable/Urgent local treatment	Less invasive, staged or symptom-focused treatment
**Neurological status**	Progressive deficit, threatened ambulation, high-grade ESCC/MSCC, short duration of motor decline.	No neurological compromise, fixed severe deficit with very limited recovery potential, or goals limited to comfort.
**Mechanical risk**	Established fracture, SINS ≥ 13, Mirels ≥ 9, progressive collapse, mechanical pain, proximal femur/periacetabular load-transfer failure.	Stable lesion, low fracture risk, tumour-mediated pain without instability, minimal load-bearing relevance.
**Biological prognosis**	Actionable molecular target, systemic treatment response, controlled visceral disease, expected survival sufficient to benefit from durable reconstruction.	Rapid systemic progression, uncontrolled visceral disease, no active systemic options, very short expected survival.
**Functional reserve**	Good ECOG/KPS, preserved rehabilitation potential, meaningful pre-morbid independence and realistic mobilisation target.	Severe frailty, poor pulmonary reserve, prohibitive perioperative risk, limited rehabilitation potential.
**Treatment goals**	Prevent irreversible neurological/mechanical decline; maintain ambulation; enable systemic therapy; restore independence.	Reduce pain, simplify care, minimise hospital burden, prioritise comfort and discharge planning.

Table S9. Proposed local treatment modality framework for bone metastases from lung cancer. Summary of major local treatment options according to best-fit indication, expected benefit and principal limitation.

Based on the findings of this systematic review and informed, where applicable, by BOA/BOOS, NICE NG234 and ESMO guidance and related orthopaedic oncology frameworks [49–52]. The figure is a conceptual, guideline-informed framework; its sequencing reflects author synthesis and it has not been prospectively validated as a prescriptive algorithm.

LOCAL TREATMENT MODALITY FRAMEWORK FOR BONE METASTASES FROM LUNG CANCER

Modality	Best-fit clinical indication	Expected benefit	Key limitation
**Open decompression and stabilisation**	Progressive neurological compromise, high-grade epidural disease with surgical fitness, unstable spine or mechanical collapse.	Neurological rescue and structural competence.	Higher physiological burden and no guaranteed survival benefit.
**Appendicular fixation / arthroplasty / endoprosthetic reconstruction**	Established fracture, impending fracture, proximal femur disease, major cortical destruction or load-bearing failure.	Early weight-bearing and durable limb function.	Must avoid relying on union when biology and RT make healing uncertain.
**Vertebral augmentation**	Selected painful vertebral lesions without major epidural compression or gross instability.	Rapid pain relief and reinforcement.	Does not decompress neural structures or correct major deformity.
**Ablation plus osteoplasty/cementoplasty**	Painful, contained or oligoprogressive lesions; frail patients; previously irradiated sites; selected pelvic or long-bone lesions.	Minimally invasive palliation and local control.	Cement leakage, embolic risk and limited role in gross instability.
**Hybrid or combined local strategies**	Complex lesions requiring simultaneous neurological, mechanical and local tumour-control objectives.	Tailored balance of stability, local control and palliation.	Requires MDT coordination and careful proportionality assessment.
**External beam RT / SBRT**	Tumour-mediated pain, local control, postoperative treatment, selected stable lesions, MSCC when surgery unsuitable or after decompression.	Effective pain/local control modality.	Does not restore mechanical stability or load transfer.
**Bone-modifying agents**	Systemic skeletal morbidity reduction in appropriate oncological context.	Reduce skeletal-related events.	Require renal, calcium, dental, MRONJ safety checks and do not treat established mechanical failure.
**Surveillance and supportive care**	Asymptomatic stable lesions, very limited reserve, comfort-focused goals or low-risk disease.	Avoids overtreatment.	Requires clear escalation triggers for new pain, mechanical symptoms or neurology.
